# Three hydrophobic amino acids in *Escherichia coli *HscB make the greatest contribution to the stability of the HscB-IscU complex

**DOI:** 10.1186/1471-2091-12-3

**Published:** 2011-01-26

**Authors:** Anna K Füzéry, Jenny J Oh, Dennis T Ta, Larry E Vickery, John L Markley

**Affiliations:** 1 Department of Biochemistry, University of Wisconsin, Madison, WI 53706, USA; 2 Department of Physiology and Biophysics, University of California, Irvine, CA 92697, USA; 3 National Magnetic Resonance Facility at Madison, University of Wisconsin, Madison, WI 53706, USA

## Abstract

**Background:**

General iron-sulfur cluster biosynthesis proceeds through assembly of a transient cluster on IscU followed by its transfer to a recipient apo-protein. The efficiency of the second step is increased by the presence of HscA and HscB, but the reason behind this is poorly understood. To shed light on the function of HscB, we began a study on the nature of its interaction with IscU. Our work suggested that the binding site of IscU is in the C-terminal domain of HscB, and two different triple alanine substitutions ([L92A, M93A, F153A] and [E97A, E100A, E104A]) involving predicted binding site residues had detrimental effects on this interaction. However, the individual contribution of each substitution to the observed effect remains to be determined as well as the possible involvement of other residues in the proposed binding site.

**Results:**

In the work reported here, we used isothermal titration calorimetry to characterize the affinity of single alanine HscB mutants for IscU, and subsequently confirmed our results with nuclear magnetic resonance spectroscopy. Alanine substitutions of L92, L96, and F153 severely impaired the ability of HscB to form a complex with IscU; substitutions of R87, R99, and E100 had more modest effects; and substitutions of T89, M93, E97, D103, E104, R152, K156, and S160 had only minor or no detectable effects.

**Conclusions:**

Our results show that the residues of HscB most important for strong interaction with IscU include three hydrophobic residues (L92, L96, and F153); in addition, we identified a number of other residues whose side chains contribute to a lesser extent to the interaction. Our results suggest that the triple alanine substitution at HscB positions 92, 96, and 153 will destabilize the HscB-IscU complex by ΔΔ*G*_b_≅ 5.7 kcal/mol, equivalent to a ≅ 15000-fold reduction in the affinity of HscB for IscU. We propose that this triple mutant could provide a more definitive test of the functional importance of the HscB-IscU interaction in vivo than those used previously that yielded inconclusive results.

## Background

Proteins containing iron-sulfur clusters play essential roles in electron-transfer, catalysis and other biochemical processes [1,2]. In eubacteria and in many eukaryotes, general iron-sulfur cluster biosynthesis is mediated by the multi-component ISC assembly system. Extensive biochemical and genetic studies [3-9] have shown that this process occurs through the assembly of a cluster on the scaffold protein IscU (Isu in yeast) followed by its transfer to a recipient apo-protein. The efficiency of the second step is greatly increased in the presence of HscA and HscB (Ssq1 and Jac1, respectively, in yeast), but the precise role of this chaperone system is not well understood [9-12].

HscB is a 20 kDa J-type co-chaperone protein that regulates the ATP hydrolysis activity of HscA and targets IscU to its substrate-binding domain. The crystal structures of HscB from *Escherichia coli *[13], *Homo sapiens *[14], and *Vibrio cholerae *[Osipiuk, Gu, Papazisi, Anderson, and Joachimiak unpublished data, PDB ID: 3HHO] revealed that the cochaperone has a conserved structural core that consists of two domains arranged in an L-shaped fold. The N-terminal J-domain (residues 1-75) is similar in structure to other J-domain fragments [15-18] while the C-terminal domain (residues 84-171) adopts a compact three-helix bundle. Sequence analysis of HscB homologs identified a series of highly conserved residues (L92, M93, L96, E97, E100, E104, and F153 in *E. coli *HscB) that form an extensive, surface-exposed patch on one face of the C-terminal domain and it was hypothesized that this region participates in functionally relevant protein-protein interactions [13].

In the cell, the physiologically relevant interaction of HscB with IscU is presumed to occur when FeS clusters are bound to the scaffold protein, e.g., IscU[2Fe2S] [9-12]. However, HscB interacts with both the apo- and holo-forms of IscU [19], and because of the instability of cluster-bound forms of IscU, studies of the HscB-IscU interaction have employed the apo-protein. The importance of several conserved residues (corresponding to L96, R99, E100, D103, E104, and Q107 in *E. coli *HscB) was first tested by collectively replacing them with alanines in *Saccharomyces cerevisiae *Jac1 [20]. The resultant hexa-mutant showed reduced affinity for Isu1 in vitro, but the authors did not identify specific residues responsible for this drop in affinity. Moreover, the hexa-mutant did not produce a growth phenotype in yeast, which suggested that either the Jac1-Isu1 interaction was not sufficiently disrupted or that it is not essential under certain conditions in vivo.

More recently, we used NMR spectroscopy and mutagenesis techniques to study the *E. coli *HscB-IscU interaction [21]. On the basis of these results, we proposed that the IscU binding site on HscB consists of the highly conserved surface patch noted previously plus nearby residues that either are conserved only in γ-proteobacteria(R99, D103, R152, K156, S160) or are not conserved (R87, T89). Triple alanine substitution of E97, E100, and E104 resulted in a modest (≅1.2-fold) decrease in the maximal synergistic stimulation of HscA by HscB and IscU and also in a modest (≅2-fold) increase in the concentration of IscU required for half-maximal stimulation. On the other hand, triple alanine substitution of L92, M93, and F153 had a much more detrimental effect: the maximal synergistic stimulation of HscA by HscB and IscU decreased ≅5-fold, and the concentration of IscU required for half-maximal stimulation increased ≅8-fold.

These studies left several important questions unanswered. Which of the alanine substitutions are responsible for the detrimental effects reported in our previous study and are there additional residues that are critical for the stability of the HscB-IscU complex? Is, in fact, the HscB-IscU interaction unnecessary under certain conditions as suggested by the Jac1-Isu1 study? In the work reported here, we used ITC and NMR spectroscopy to define the individual contribution of fourteen HscB residues to the stability of the HscB-(apo-IscU) complex. Our results allowed us to refine the residues of HscB critical for strong interaction with IscU to three hydrophobic residues (L92, L96, and F153), with a single negatively charged residue (E100), and two positively charged residues (R87 and R99) providing additional but smaller contributions. Moreover, we suggest a pattern of alanine substitution that could prove useful in testing the importance of the HscB-IscU interaction in vivo.

## Results

### Structure and stability of alanine-substituted HscBs

Using the QuikChange technique and previously described expression and purification procedures [19,21,22], we generated single alanine substitutions in *E. coli *HscB at positions 87, 89, 92, 93, 96, 97, 99, 100, 103, 104, 152, 153, 156, and 160. All of the mutants displayed far- and near-UV CD spectra similar to wild-type HscB (see Additional File 1). In addition, mutants with an alanine substitution at position 92, 93, 96, 97, 99, 153, or 156 displayed solution molecular masses (≅29 kDa) and thermal stabilities (*T*_m_≅ 65°C) similar to wild-type HscB (see Additional Files 2 and 3, respectively). Although HscB(R152A) also displayed a solution molecular mass ≅29 kDa, it showed a slightly lower melting temperature (*T*_m_≅ 62°C) than wild-type HscB (see Additional Files 2 and 3, respectively). Together, these results suggest that the overall structure, oligomeric state, and stability of HscB are not affected to any significant degree by the single alanine substitutions.

### Affinity of alanine-substituted HscBs for IscU

**Figure 1:**
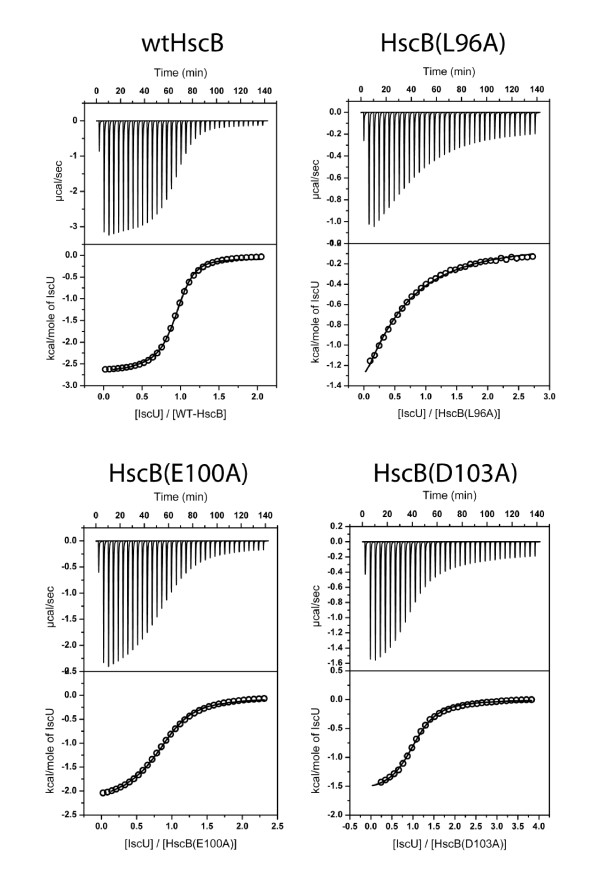
**ITC data for titration of wild-type or selected alanine-substituted forms of HscB with apo-IscU**. Titrations were performed in 50 mM HEPES pH 7.5, 150 mM NaCl, 4 mM TCEP at 25 °C. The concentrations of reactants were 0.25-0.4 mM HscB and 2.5-4 mM apo-IscU. Additional File 4 contains similar data for the remaining alanine-substituted forms of HscB.

To investigate whether any of the alanine substitutions perturbed the stability of the HscB-IscU complex, we used ITC to determine the binding affinity of each alanine mutant for apo-IscU (Table 1, Figure 1, and Additional File 4). Under the conditions of the experiment, wild-type HscB bound IscU with an affinity of 9 ± 2 μM which approximates the previously reported value of 13 μM [19]. Alanine substitutions at position 89, 93, 97, 103, 104, 152, 156, or 160 had no or only minor effects on the binding of HscB to IscU (*K*_d _≅ 4-17 μM). In contrast, substitutions R87A (*K*_d _≅ 36 μM), R99A (*K*_d _≅ 53 μM), and E100A (*K*_d _≅ 42 μM) decreased the affinity of HscB for IscU ≅ 4- to 6-fold. The most dramatic effects were observed for three hydrophobic residues in the center of the proposed IscU binding site. Substitutions L92A (*K*_d _≅ 200 μM), L96A (*K*_d _≅ 200 μM), and F153A (*K*_d _≅ 200 μM) decreased the affinity of HscB for IscU greater than 20-fold compared to that of wild-type protein.

**Table 1:** ITC binding isotherms for the interaction of IscU with wild-type and alanine-substituted forms of HscB^1^

HscB	*n*	*K*_a_(10^5^M^-1^)	Δ*H*(kcal/mol)	Δ*S *(e.u.)
wild-type	1.02 ± 0.08	1.16 ± 0.28	-2.22 ± 0.34	15.7 ± 0.7

R87A	1.04	0.28	-0.88	17.4

T89A	1.08	0.59	-1.00	18.4

L92A	0.70	0.05	0.41	18.3

M93A	0.83 ± 0.04	2.55 ± 0.49	-1.25 ± 0.07	20.4 ± 0.1

L96A	0.44 ± 0.04	0.05 ± 0.01	-3.60 ± 0.00	4.8 ± 0.6

E97A	0.96	1.73	-2.60	15.2

R99A	0.69	0.19	1.18	23.6

E100A	0.91	0.24	-2.22	12.0

D103A	1.04	0.54	-1.61	16.2

E104A	0.83	1.72	-2.80	14.6

R152A	1.09	0.65	-1.34	17.5

F153A	0.78	0.05	0.58	19.0

K156A	0.93	2.59	-2.64	15.9

S160A	1.05	0.92	-1.58	17.4

E97A, E100A, E104A	0.68	0.33	-1.73	14.9

^1^Stoichiometries *(n)*, association constants (*K*_a_), heats of binding (Δ*H*), and entropies of binding (Δ*S*) for the interaction of wild-type and alanine-substituted forms of HscB with apo-IscU, as determined by ITC. The entropic contribution was determined from the relationship, ΔG_b _= ΔH_b _- TΔS_b_, where ΔG_b _= -RTlnK_a_. Owing to the low *n *values obtained with HscB(L92A), HscB(L96A), HscB(R99A), HscB(F153A), and HscB(E97A, E100A, E104A), the ΔH and Δ*S *values determined for these variants are likely only approximate. Gb values cited in the text were calculated from the relationship, ΔΔG_b _= ΔG_b _(mutant HscB) - ΔG_b _(wild-type HscB).

To investigate the effect of multiple alanine substitutions on the affinity of HscB for IscU, we also evaluated by ITC the behaviour of our previously studied triple alanine mutants. The (E97A, E100A, E104A) substitution had a modest effect on the binding of HscB to IscU (*K*_d _≅ 30 μM), and this change was almost equal to that observed for the E100A single mutant. In contrast, the effect of the (L92A, M93A, F153A) substitution was much greater than the effect of any single alanine substitution. The heats of injection for this triple mutant were very small (less than 0.2 kcal/mol), and fitting of the data to extract binding parameters was unsuccessful.

### NMR spectroscopy of alanine-substituted HscB

As an independent means of assessing the effects of the single alanine substitutions, we used NMR spectroscopy to compare selected HscB mutants with wild-type HscB. One representative mutant was chosen from each of the three types of mutants observed in our ITC studies: HscB(D103) (little or no effect on affinity of HscB for IscU), HscB(E100A) (modest effect on affinity of HscB for IscU), and HscB(L96A) (dramatic effect on affinity of HscB for IscU). The ^15^N-HSQC spectra of the free proteins were very similar (see Additional Files 5, 6, 7, 8, 9, 10), and larger chemical shift differences (≅ 0.040 ppm) were only observed for residues in the immediate vicinity of the alanine substitutions (F91-K109). The presence of a six-fold molar excess of IscU produced larger chemical shift differences among the ^15^N-HSQC spectra of the proteins, but the overall peak pattern of all four spectra remained very similar (see Additional File 11). Together, these results indicate that an alanine substitution at position 96, 100 or 103 does not alter the overall fold of free HscB to any significant degree and that the different forms of HscB adopt similar structures in the HscB-IscU complex.

Next, we recorded a series of ^15^N-HSQC spectra for HscB in the presence of varying amounts of apo-IscU. We observed previously that many of the NMR signals that map to the IscU binding site on HscB are broad and unobservable in the HscB-IscU complex [21]. Thus those HscB signals observed to shift upon addition of IscU need not all be in the contact region and may simply report on a conformational transition that accompanies IscU binding. Nevertheless, their chemical shift changes report on the binding interaction and can be used to assess binding strength. Therefore, we reasoned that if a substitution leaves the HscB-IscU binding strength unperturbed, the complex will be saturated to a similar extent at any given HscB/IscU ratio relative to the wild-type HscB-IscU complex. In turn, this will cause the progressive IscU-induced chemical shift changes exhibited by the mutant HscB to be very similar to those of wild-type HscB. Based on our ITC results, we hypothesized that HscB(D103A) will show this type of behaviour. In contrast, if a substitution decreases the HscB-IscU binding strength, the complex should be saturated to a smaller extent at any given HscB/IscU ratio relative to the wild-type HscB-IscU complex. In turn, this will cause the progressive IscU-induced chemical shift changes exhibited by the mutant HscB to be smaller than those of wild-type HscB. Based on our ITC results, we hypothesized that HscB(L96A) and HscB(E100A) will show this type of behaviour.

**Figure 2:**
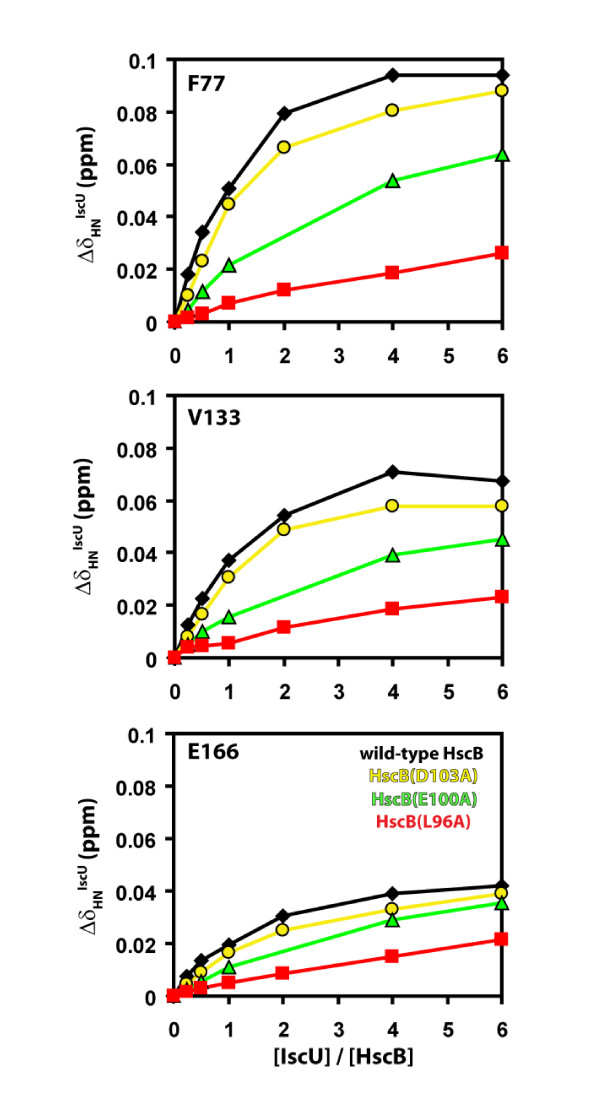
**NMR data for the titration of [*U*-^15^N]-labeled HscB with apo-IscU**. Combined chemical shift changes (Δδ_HN_^IscU^) of selected residues are plotted as a function of IscU/HscB molar ratio for wild-type HscB (black diamonds), HscB(D103A) (yellow circles), HscB(E100A) (green triangles), and HscB(L96A) (red squares). Δδ_HN_^IscU ^values are reported as the combination of changes in the proton (H^IscU^) and nitrogen (Δδ_N_^IscU^) dimensions according to Δδ_HN_^IscU ^= [(Δδ_H_^IscU^)^2 ^+ Δδ_N_^IscU^/6)^2^]^1/2 ^[29]. Δδ_H_^IscU ^and Δδ_N_^IscU ^are calculated relative to the free form of each protein.

The ^15^N-HSQC spectral series for each mutant was first examined to identify all peaks showing chemical shift changes during the titration; subsequently, the observed chemical shift changes were plotted as a function of IscU/HscB molar ratio, and the results were compared to those obtained for wild-type HscB. Figure 2 shows examples of such a comparison for three well resolved peaks in the HSQC spectral series: F77, V133, and E166. The behaviour of F77 is representative of the majority of peaks that experienced large chemical shift changes, while the behaviours of V133 and E166 are representative of the majority of peaks that experienced moderate and small chemical shift changes, respectively.

For HscB(D103A), F77, V133, and E166 showed a very similar pattern of progressive chemical shift changes to that observed for wild-type HscB (yellow circles versus black diamonds). This indicates that at any given HscB/IscU ratio, the complex is similarly saturated whether it contains wild-type HscB or HscB(D103A). Therefore, an alanine substitution at position 103 of *E. coli *HscB affects the protein's affinity for IscU only slightly. For HscB(E100A), the progressive chemical shift changes for all three residues appeared ≅ 25-50% smaller than for wild-type HscB at each HscB-IscU molar ratio examined (green triangles versus black diamonds). This indicates that at any given HscB/IscU ratio, the complex containing the mutant is less saturated than that containing the wild-type protein. Therefore, an alanine substitution at position 100 of *E. coli *HscB affects its affinity for IscU to a greater extent than the D103A substitution. Of the three alanine substitutions examined, the one at position 96 had the largest effects. The progressive chemical shift changes for F77, V133, and E166 of HscB(L96A) were over 50% smaller than for wild-type HscB at each HscB-IscU molar ratio examined (red squares versus black diamonds). This indicates that at any given HscB/IscU ratio, the complex containing the L96A mutant is much less saturated than that containing the wild-type protein or either of the other two mutants. Therefore, an alanine substitution at position 96 of *E. coli *HscB produces the largest decrease in the affinity of the cochaperone for IscU.

**Figure 3:**
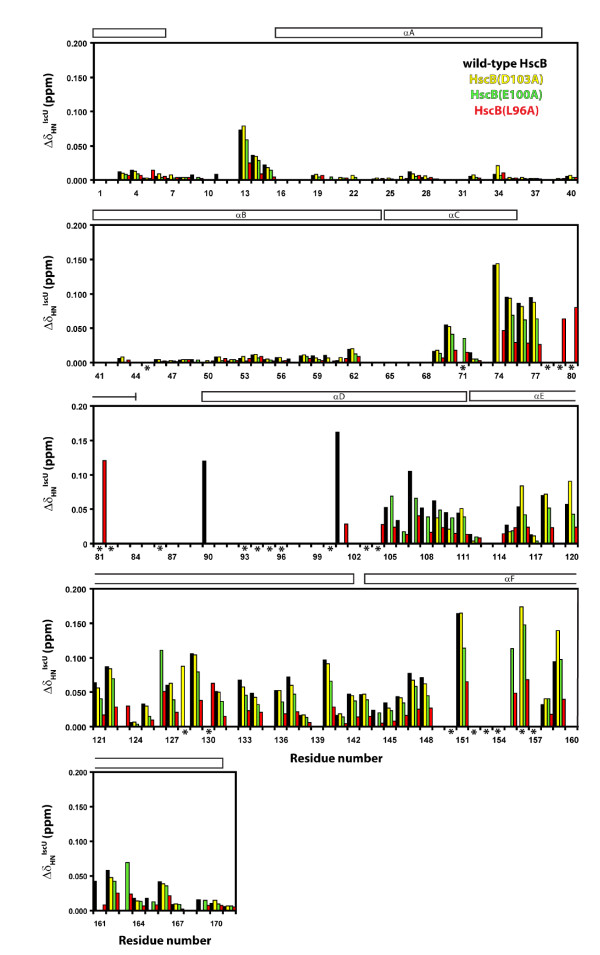
**NMR data for [*U*-^15^N]-labeled HscB in the presence of a six-fold molar excess of apo-IscU**. Combined chemical shift changes (Δδ_HN_^IscU^) are plotted for wild-type HscB (black), HscB(D103A) (yellow), HscB(E100A) (green), and HscB(L96A) (red). Δδ_HN_^IscU ^values are reported as the combination of changes in the proton (Δδ_H_^IscU^) and nitrogen (Δδ_N_^IscU^) dimensions according to Δδ_HN_^IscU ^= [(Δδ_H_^IscU^)^2 ^+ Δδ_N_^IscU^/6)^2^]^1/2 ^[29]. Δδ_H_^IscU ^and Δδ_N_^IscU ^are calculated relative to the free form of each protein. Residues whose peaks disappear in the presence of apo-IscU for at least one form of HscB are marked with an asterisk and are also listed in Additional Files 5 to 8. Values are not shown for residues that have an unassigned ^1^H^N^-^15^N cross-peak in free HscB, that lack an observable ^1^H-^15^N cross-peak in free and/or bound HscB, have an overlapped ^1^H-^15^N cross-peak in free and/or bound HscB, or are prolines (P10, P33, and P64). The diagram at the top of each panel shows the location of secondary structural elements in *E. coli *HscB.

Figure 3 shows the combined chemical shift changes observed for all residues of wild-type HscB and of the three single alanine HscB mutants in the presence of a six-fold molar excess of apo-IscU. The pattern of chemical shift changes parallels that observed for F77, V133, and E166: residues of HscB(D103A) behave similarly to wild-type HscB (yellow versus black bars), residues of HscB(E100A) show changes that are ≅ 10-25% smaller than for wild-type HscB (green versus black bars), and residues of HscB(L96A) show changes that are over 50% smaller than for wild-type HscB (red versus black bars). Therefore, these results also support the conclusion that the D103A substitution has the smallest effect on the affinity of HscB for IscU whereas the L96A substitution has the largest effect. Taken together, the NMR results are fully consistent with the ITC data and confirm that alanine substitutions at different positions within the proposed binding site affect the affinity of HscB for IscU to different degrees.

## Discussion

**Figure 4:**
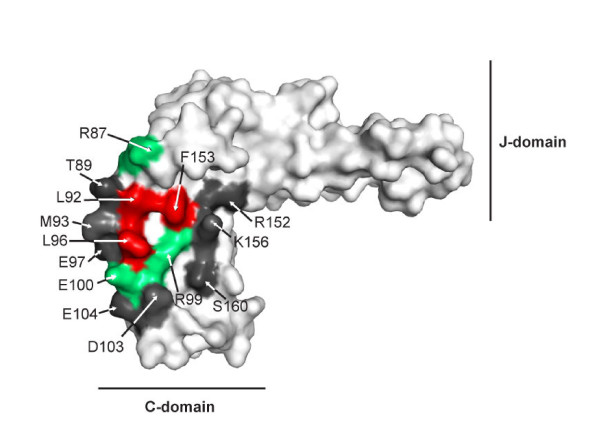
**ITC results mapped onto the crystal structure of *E. coli *HscB**. Alanine substitution of L92, L96, or F153 (red) had the most detrimental effects on the affinity of HscB for apo-IscU, while alanine substitution at positions 87, 99, or 100 (green) resulted in smaller effects. Alanine substitution at positions 89, 93, 97, 103, 104, 152, 156, or 160 (dark grey) left the affinity of HscB for apo-IscU unchanged. This image was prepared with PyMol [30].

The experiments described herein continue previous work from our laboratory on the HscB-IscU interaction. We individually replaced with alanine fourteen surface-exposed residues in the C-terminal domain of *E. coli *HscB, and evaluated their effects using ITC and NMR spectroscopy. Of the three highly conserved acidic residues (E97, E100, E104), only the E100A substitution perturbed the affinity of HscB for IscU (Figure 4). The resultant change in the free energy of binding (ΔΔ*G*_b _≅ 0.9 kcal/mol) was close to the experimentally determined value for a mutant with alanine substitutions at all three acidic positions, suggesting that the effects of the substitutions are additive and that the slightly decreased function of the triple mutant observed in our previous work [21] is caused predominantly by the E100A substitution.

By contrast, of the four highly conserved hydrophobic residues of HscB, three make major contributions to the stability of the complex with apo-IscU (Figure 4). Alanine substitutions at positions 92, 96, or 153 decreased the affinity of HscB for IscU ≅ 5-fold more than the E100A substitution, and resulted in ΔΔ*G*_b _values of approximately 1.9 kcal/mol. Although L92, L96, and F153 appear to be the most important residues, others (T87, R99, E100) were also found to make substantial contributions to the stability of the HscB-IscU complex. On the other hand, substitutions at positions 89, 93, 97, 103,104, 156, and 160 had little or no effect; this suggests that these residues are located at the periphery of the HscB-IscU interface where additional solvent molecules and the rearrangement of hydrogen bonding patterns can more easily accommodate a missing side chain [23,24].

## Conclusions

The present results explain earlier studies of the triple mutant, HscB(L92A, M93A, F153A). Of these three substitutions, L92A and F153A are the ones expected to affect the interaction with IscU, and we can estimate that they will destabilize the HscB-IscU complex by ≅ 3.7 kcal/mol. This large effect explains the very small (less than 0.2 kcal/mol) heats of injection observed in our ITC experiments (Additional File 4), the low level of HscA stimulation achieved by this mutant [21], and the weak NMR shifts of ^15^N-labeled IscU when titrated with this mutant [25].

Our results also suggest a new pattern of alanine substitution to test the in vivo significance of the HscB-IscU interaction. If the effect of each substitution is additive, alanine substitutions at positions 96, 99, 100, 103, 104, and 107 (corresponding to the hexa-alanine substitution made in Jac1 previously [20]) will destabilize the HscB-IscU complex by ≅ 4.2 kcal/mol, corresponding to a ≅ 1000-fold reduction in the affinity of HscB for IscU. While this is a large drop in affinity, it might not be sufficient to completely disrupt the HscB-IscU interaction which could explain why a growth phenotype was not seen for the corresponding Jac1 mutant. Our data suggest, however, that a triple alanine substitution at HscB positions 92, 96, and 153 will destabilize the HscB-IscU complex to a much greater extent (ΔΔ*G*_b _≅ 5.7 kcal/mol), corresponding to a ≅ 15000-fold reduction in affinity of HscB for IscU. This triple alanine substitution is expected to disrupt the HscB-IscU interaction more completely than the hexa-alanine substitution described above and could prove useful for in vivo testing of the functional significance of the HscB-IscU interaction.

## Methods

### Site-directed mutagenesis

Mutations in vectors for expressing mutants of HscB were generated using the QuikChange technique and were confirmed by DNA sequencing.

### Protein sample preparation

HscB and IscU proteins were made as described previously [19,21,22]. Briefly, HscB and IscU were expressed in *Escherichia coli *grown in TB media or in M9 minimal media containing ^15^NH4Cl. To extract and purify HscB, cells were lysed by sonication in TED buffer (50 mM Tris-HCl, pH 7.4, 1 mM DTT, 0.5 mM EDTA) and the soluble fraction was applied to a room temperature DEAE-cellulose column (DE-52; Whatman). HscB was eluted using a linear gradient from 1 to 100 mM NaCl, followed by an isocratic elution with 100 mM NaCl. Fractions found to contain HscB were pooled, concentrated, and further fractionated using size exclusion chromatography. Homogeneous fractions of HscB were pooled, concentrated, frozen in liquid nitrogen, and stored at 80 °C. To extract and purify IscU, cells were lysed by sonication in TED8 buffer (50 mM Tris-HCl, pH 8, 1 mM DTT, 0.5 mM EDTA) and the soluble fraction was purified at 4 °C by ion-exchange chromatography with DEAE-cellulose, DEAE-Biogel (Bio-Rad), and elution gradients of 0 to 150 mM NaCl. Fractions found to contain IscU were pooled, concentrated, and further fractionated at room temperature using size exclusion chromatography. Homogeneous fractions of IscU were pooled, concentrated, frozen in liquid nitrogen, and stored at 80 °C.

### Molecular mass determination

The molecular size of wild-type and alanine-substituted HscBs was estimated by calibrated gel filtration. Chromatography was carried out at 15 °C using a BioSep-SEC-S 2000 column (Phenomenex) connected to a Shimadzu UFLC system. The column was first equilibrated in 50 mM Tris-HCl pH 7.6, 150 mM NaCl; subsequently a 1 μL aliquot of sample was applied and eluted using the same buffer. Total protein concentration for all injected samples was ≅ 1 mg/mL. Standards used included ferritin (440 kDa), ovalbumin (43 kDa), carbonic anhydrase (29 kDa), and aprotinin (6.5 kDa). All samples were prepared in equilibration buffer. *K*_ave _values were calculated according to *K*_ave _= (*V*_e _- *V*_o_) / (*V*_c _- *V*_o_), where *V*_o _is the column void volume as determined with Blue Dextran, *V*_c _is the geometric column volume and *V*_e _is the elution volume of the molecule under consideration. All runs were carried out in duplicate.

### CD spectroscopy

CD studies were performed on an AVIV 202SF Stopped Flow Circular Dichroism Spectrometer equipped with a multi-cell holder. Far-UV spectra were collected from 195 to 300 nm in 1 nm increments with an averaging time of 5 s, and used ≅ 7 μM protein in 20 mM Tris-H2SO4 pH 8.0 buffer and a 1 mm path length cell. Near-UV spectra were collected from 250 to 340 nm in 0.5 increments with an averaging time of 10 s, and used≅ 41 μM protein in 20 mM Tris-HCl pH 7.9, 1 mM DTT buffer and a 10 mm path length cell. All spectra were collected at 25 °C and represent a single scan with the buffer baseline subtracted.

Temperature-induced unfolding of wild-type and alanine-substituted forms of HscB were carried out on samples containing ≅ 8 μM protein in 50 mM sodium phosphate pH 7.5 buffer, and were monitored at 200 nm, 208 nm, and 222 nm in a 1 mm path length cell. The temperature was cycled from 25 °C to 85 °C in increments of 3 °C. Prior to taking a reading, a 5 minute thermal equilibration period was established at each newly set temperature. Once data acquisition at 85 °C was complete, samples were cooled to 25 °C and a far-UV spectrum was collected for each. The pre- and post-melt spectra were almost identical for every mutant examined, indicating reversibility of unfolding. Denaturation profiles were analyzed by a nonlinear least-squares fit assuming a two state model [26] and with Δ*C*_p _fixed at 0. Resultant *T*_m _and Δ*H*_m _values were averaged over the three wavelengths at which data were collected as well as over a duplicate set of experiments.

### ITC

ITC measurements were carried out using a VP-ITC isothermal titration calorimeter (MicroCal LLC). Titrations were performed at 25 °C in 50 mM HEPES pH 7.5, 150 mM NaCl, 4 mM TCEP. Samples contained 0.25-0.4 mM HscB (sample cell) and 2.5-4 mM apo-IscU (injection syringe). Solutions were thoroughly degassed by stirring under a vacuum before use. Each titration was started with an initial injection of 3 μL, followed by 34 injections of 8 μL with a 240 second gap between consecutive injections. The peaks of the obtained thermograms were integrated using the ORIGIN software supplied with the instrument. A nonlinear regression fit to each isotherm was performed using the ORIGIN data analysis routine, yielding binding constants (*K*_a_), enthalpies of binding (Δ*H*), and stoichiometry (*n*). Data points from the first injections were normally removed prior to the fitting process. Heat produced upon injection of buffer into buffer was small (less than 0.2 kcal/mol), and the data presented in the text are not corrected for this effect. For all experiments except those involving HscB(L92A), HscB(L96A), HscB(R99A), and HscB(F153A), the heat of dilution of apo-IscU was approximated using the heats of the last few injections. For HscB(L92A), HscB(L96A), HscB(R99A), and HscB(F153A), saturation did not appear to be complete by the end of the experiment. Therefore, the heat of dilution of apo-IscU was obtained from a separate experiment in which apo-IscU was titrated into buffer using the injection schedule described above. Errors from the fitting procedure were always smaller than 0.04 (*n*), 0.50 (*K*_a_), and 0.40 (Δ*H*). All experiments were performed once except for wild-type HscB (three repetitions), HscB(M93A) (two repetitions), and HscB(L96A) (two repetitions). Average values and standard deviations are provided for these three forms of HscB.

### NMR spectroscopy

Samples for chemical shift perturbation studies contained 0.2 mM [*U*-^15^N]-HscB and varying concentrations of unlabelled apo-IscU (0 mM, 0.05 mM, 0.1 mM, 0.2 mM, 0.4 mM, 0.8 mM, or 1.2 mM) in 20 mM Tris-HCl pH 7.5, 10 mM DTT, 0.1 mM DSS, and 7 % D2O. A 2D ^15^N-HSQC was recorded for each sample at 40 °C on a Bruker DMX-750 Avance spectrometer equipped with a *z*-axis gradient cryoprobe. All spectra were processed with NMRPipe [27] and analyzed with Sparky [28]. Amide ^1^H and ^15^N resonances for the mutant proteins were assigned with the help of our previously determined assignments for wild-type HscB [21]. Changes in amide peak positions of HscB as a result of the alanine substitutions, Δδ_HN_^Ala ^(in ppm), are reported as a combination of the changes in the proton (Δδ_H_^Ala^) and nitrogen (Δδ_N_^Ala^) dimensions according to Eq. 1 [29].

(1) $$\Delta \delta_{HN}{}^{\text{Ala}}={\left[{\left(\Delta \delta_{\text{H}}{}^{\text{Ala}}\right)}^{\text{2}}+{\left(\Delta \delta_{\text{N}}{}^{\text{Ala}}\text{/6}\right)}^{\text{2}}\right]}^{\text{1}/\text{2}}\;$$

Apo-IscU-induced changes in the amide peak position of HscB, Δδ_HN_^IscU^, were also calculated according to Eq. 1 but using Δδ_H_^IscU ^and Δδ_N_^IscU ^instead of Δδ_H_^Ala ^and Δδ_N_^Ala^.

## List of abbreviations used

NMR: nuclear magnetic resonance; ITC: isothermal titration calorimetry; CD: circular dichroism; [*U*-^15^N], uniformly ^15^N-labeled

## Authors' contributions

AKF, JLM, and LEV participated in the design of the study and in the draft of the manuscript. AKF, JJO, and DTT carried out the experiments described in the text. All authors read and approved the final manuscript.

## Acknowledgements

This work was supported by NIH grants GM58667 (to J.L.M.) and GM54264 (to L.E.V.). CD and ITC data were obtained at the University of Wisconsin -- Madison Biophysics Instrumentation Facility, which was established with support from the University of Wisconsin -- Madison and grants BIR-9512577 (NSF) and S10 RR13790 (NIH). NMR data were collected at the National Magnetic Resonance Facility at Madison (NMRFAM), which is supported in part by NIH Grants P41 RR02301 and P41 GM66326. We thank Darrell McCaslin for assistance with CD and ITC data collection. We thank Karl Nichols and the Center for Eukaryotic Structural Genomics for help in carrying out the gel filtration experiments.

## Supplementary Material

Additional File 1 Far-UV and near-UV CD spectra of wild-type and alanine-substituted forms of HscB Click here for file

Additional File 2 Apparent molecular masses of wild-type and selected alanine-substituted forms of HscB, as determined by analytical gel filtration Click here for file

Additional File 3 Transition parameters for the thermal denaturation of wild-type and selected alanine-substituted forms HscB Click here for file

Additional File 4 Representative ITC binding isotherms for the interaction of IscU with wild-type and alanine-substituted forms of HscB
Titrations were performed in 50 mM HEPES pH 7.5, 150 mM NaCl, 4 mM TCEP at 25 °C. The concentrations of reactants were 0.25-0.4 mM HscB (cell) and 2.5-4 mM apo-IscU (injection syringe). Click here for file

Additional File 5 ^15^N-HSQC spectra of wild-type HscB, HscB(D103A), HscB(E100A), and HscB(L96A)
Samples contained 0.2 mM [*U*-^15^N]-HscB in 20 mM Tris-HCl pH 7.5, 10 mM DTT, 0.1 mM DSS, and 7 % D2O. Each spectrum was recorded at 40 °C on a Bruker DMX-750 Avance spectrometer equipped with a *z*-axis gradient cryoprobe. Peaks that are folded into the spectrum are colored in magenta. Click here for file

Additional File 6 Chemical shift assignments for free and IscU-bound wild-type HscB
^1^H (δ_H_) and ^15^N (δ_N_) chemical shifts (in ppm) of assigned peaks in the ^15^N-HSQC spectrum of unbound HscB, and HscB in the presence of a six-fold molar excess of IscU ("IscU-bound HscB"). Click here for file

Additional File 7 Chemical shift assignments for free and IscU-bound HscB(D103A)
^1^H (δ_H_) and ^15^N (δ_N_) chemical shifts (in ppm) of assigned peaks in the ^15^N-HSQC spectrum of unbound HscB(D103A), and HscB(D103A) in the presence of a six-fold molar excess of IscU ["IscU-bound HscB(D103A)"]. Click here for file

Additional File 8 Chemical shift assignments for free and IscU-bound HscB(E100A)
^1^H (δ_H_) and ^15^N (δ_N_) chemical shifts (in ppm) of assigned peaks in the ^15^N-HSQC spectrum of unbound HscB(E100A), and HscB(E100A) in the presence of a six-fold molar excess of IscU ["IscU-bound HscB(E100A)"]. Click here for file

Additional File 9 Chemical shift assignments for free and IscU-bound HscB(L96A)
^1^H (δ_H_) and ^15^N (δ_N_) chemical shifts (in ppm) of assigned peaks in the ^15^N-HSQC spectrum of unbound HscB(L96A), and HscB(L96A) in the presence of a six-fold molar excess of IscU ["IscU-bound HscB(L96A)"]. Click here for file

Additional File 10 Combined chemical shift changes of free HscB(D103A), HscB(E100A), and HscB(L96A) relative to wild-type HscB
Combined chemical shift changes are reported as the combination of changes in the proton (Δδ_H_^Ala^) and nitrogen (Δδ_N_^Ala^) dimensions according to Δδ_HN_^Ala ^= [(Δδ_H_^Ala^)^2 ^+ Δδ_N_^Ala^/6)^2^]^1/2 ^[29]. Click here for file

Additional File 11 ^15^N-HSQC spectra of wild-type HscB, HscB(D103A), HscB(E100A), and HscB(L96A) in the presence of a six-fold molar excess of IscU
Samples contained 0.2 mM [*U*-^15^N]-HscB and 1.2 mM IscU in 20 mM Tris-HCl pH 7.5, 10 mM DTT, 0.1 mM DSS, and 7 % D_2_O. Each spectrum was recorded at 40°C on a Bruker DMX-750 Avance spectrometer equipped with a *z*-axis gradient cryoprobe. Peaks that are folded into the spectrum are colored in magenta. Click here for file
