# Supplementary figures and images for: Three hydrophobic amino acids in Escherichia coli HscB make the greatest contribution to the stability of the HscB-IscU complex
Source: BMC Biochem. 2011 Jan 26;12:3. doi: 10.1186/1471-2091-12-3 (PMC3040723; doi:10.1186/1471-2091-12-3)

**Figure S1 – Far-UV and near-UV CD spectra of** **wild-type and alanine-substituted forms of HscB**

**
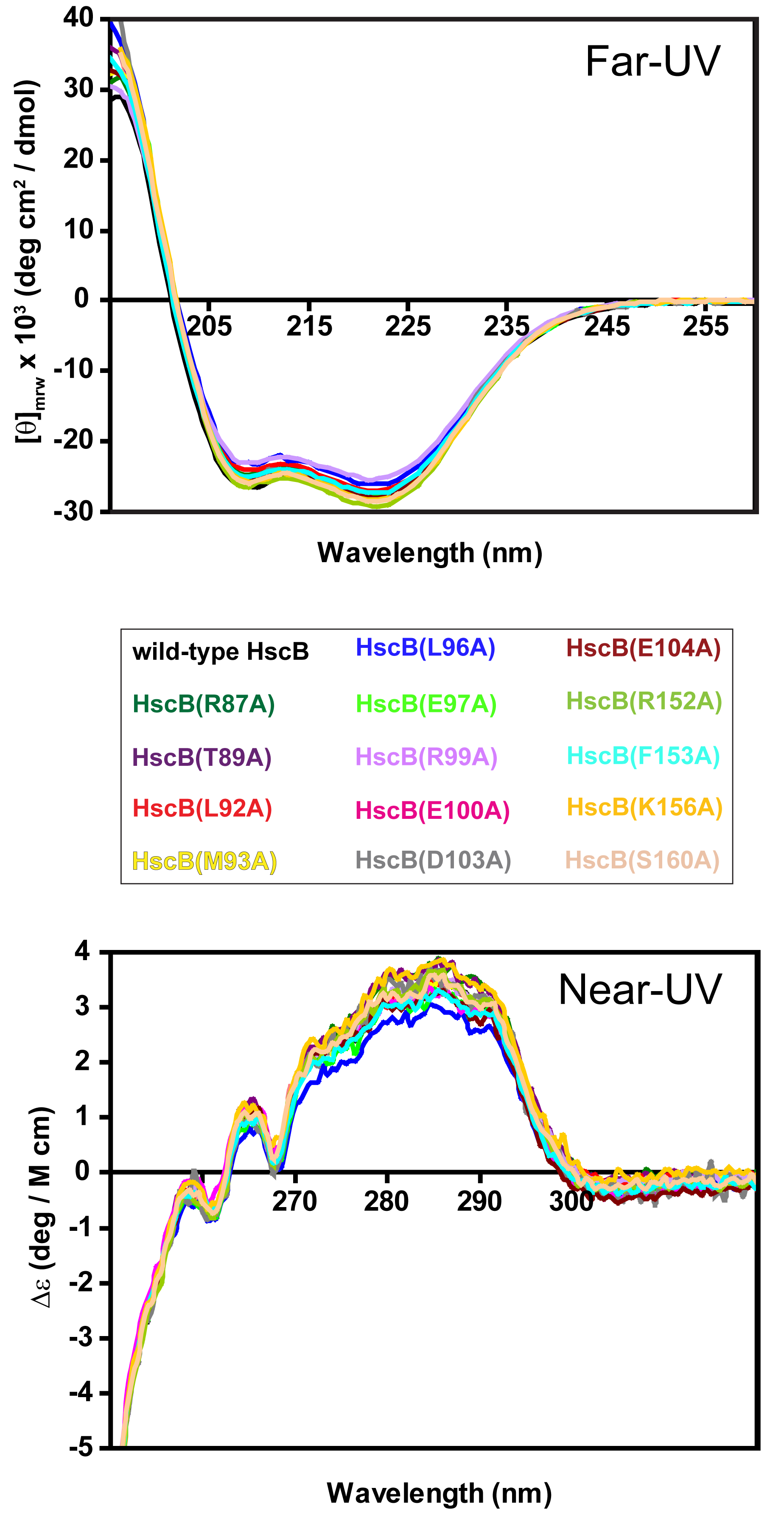
**

Supplement: Additional File 1 — Far-UV and near-UV CD spectra of wild-type and alanine-substituted forms of HscB [file 1471-2091-12-3-S1.DOC]
