# Supplementary material for: Three hydrophobic amino acids in Escherichia coli HscB make the greatest contribution to the stability of the HscB-IscU complex
Source: BMC Biochem. 2011 Jan 26;12:3. doi: 10.1186/1471-2091-12-3 (PMC3040723; doi:10.1186/1471-2091-12-3)
Supplement: Additional File 2 — Apparent molecular masses of wild-type and selected alanine-substituted forms of HscB, as determined by analytical gel filtration [file 1471-2091-12-3-S2.DOC]

**Table S1 – Apparent molecular masses of wild-type and selected alanine-substituted forms of HscB, as determined by analytical gel filtration**

| HscB | Molecular mass (kDa) |
| --- | --- |
| wild-type | 28.7 ± 0.5 |
| L92A | 29.5 ± 0.4 |
| M93A | 29.6 ± 0.5 |
| L96A | 29.5 ± 0.3 |
| E97A | 28.4 ± 0.3 |
| R99A | 30.1 ± 0.6 |
| R152A | 29.9 ± 0.4 |
| F153A | 29.6 ± 0.5 |
| K156A | 29.9 ± 0.3 |
