# Supplementary material for: Three hydrophobic amino acids in Escherichia coli HscB make the greatest contribution to the stability of the HscB-IscU complex
Source: BMC Biochem. 2011 Jan 26;12:3. doi: 10.1186/1471-2091-12-3 (PMC3040723; doi:10.1186/1471-2091-12-3)
Supplement: Additional File 3 — Transition parameters for the thermal denaturation of wild-type and selected alanine-substituted forms HscB [file 1471-2091-12-3-S3.DOC]

**Table S2 – Transition parameters for the thermal denaturation of wild-type and selected alanine-substituted forms HscB**

| HscB | *T*_m_ (°C) | Δ*H*_m_ (kcal/mol) |
| --- | --- | --- |
| wild-type | 65.3 ± 0.1 | 115 ± 4 |
| L92A | 64.8 ± 0.2 | 111 ± 8 |
| M93A | 65.6 ± 0.2 | 111 ± 8 |
| L96A | 65.8 ± 0.2 | 113 ± 6 |
| E97A | 65.9 ± 0.2 | 105 ± 3 |
| R99A | 64.8 ± 0.2 | 104 ± 3 |
| R152A | 61.8 ± 0.2 | 103 ± 3 |
| F153A | 64.4 ± 0.2 | 111 ± 6 |
| K156A | 65.7 ± 0.2 | 115 ± 7 |
