# Supplementary material for: Three hydrophobic amino acids in Escherichia coli HscB make the greatest contribution to the stability of the HscB-IscU complex
Source: BMC Biochem. 2011 Jan 26;12:3. doi: 10.1186/1471-2091-12-3 (PMC3040723; doi:10.1186/1471-2091-12-3)
Supplement: Additional File 4 — Representative ITC binding isotherms for the interaction of IscU with wild-type and alanine-substituted forms of HscB Titrations were performed in 50 mM HEPES pH 7.5, 150 mM NaCl, 4 mM TCEP at 25 °C. The concentrations of reactants were 0.25-0.4 mM HscB (cell) and 2.5-4 mM apo-IscU (injection syringe). [file 1471-2091-12-3-S4.DOC]

**Figure S2 – Representative ITC binding isotherms for the interaction of apo-IscU with wild-type and alanine-substituted forms of HscB**

Titrations were performed in 50 mM HEPES pH 7.5, 150 mM NaCl, 4 mM TCEP at 25 ºC. The concentrations of reactants were 0.25-0.4 mM HscB and 2.5-4 mM apo-IscU.

**Figure S2 (continued)**

**
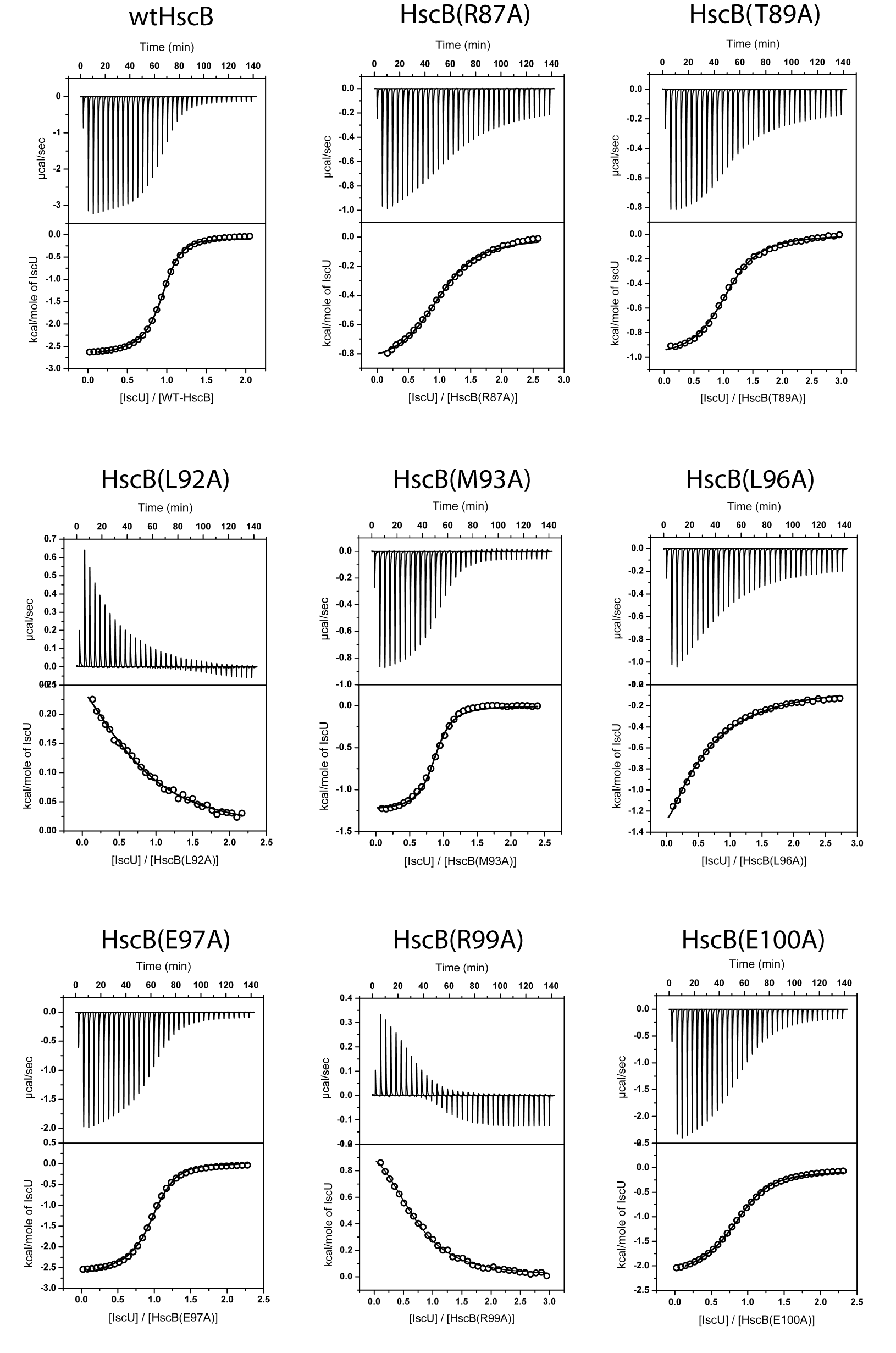
**

**Figure S2 (continued)**

**
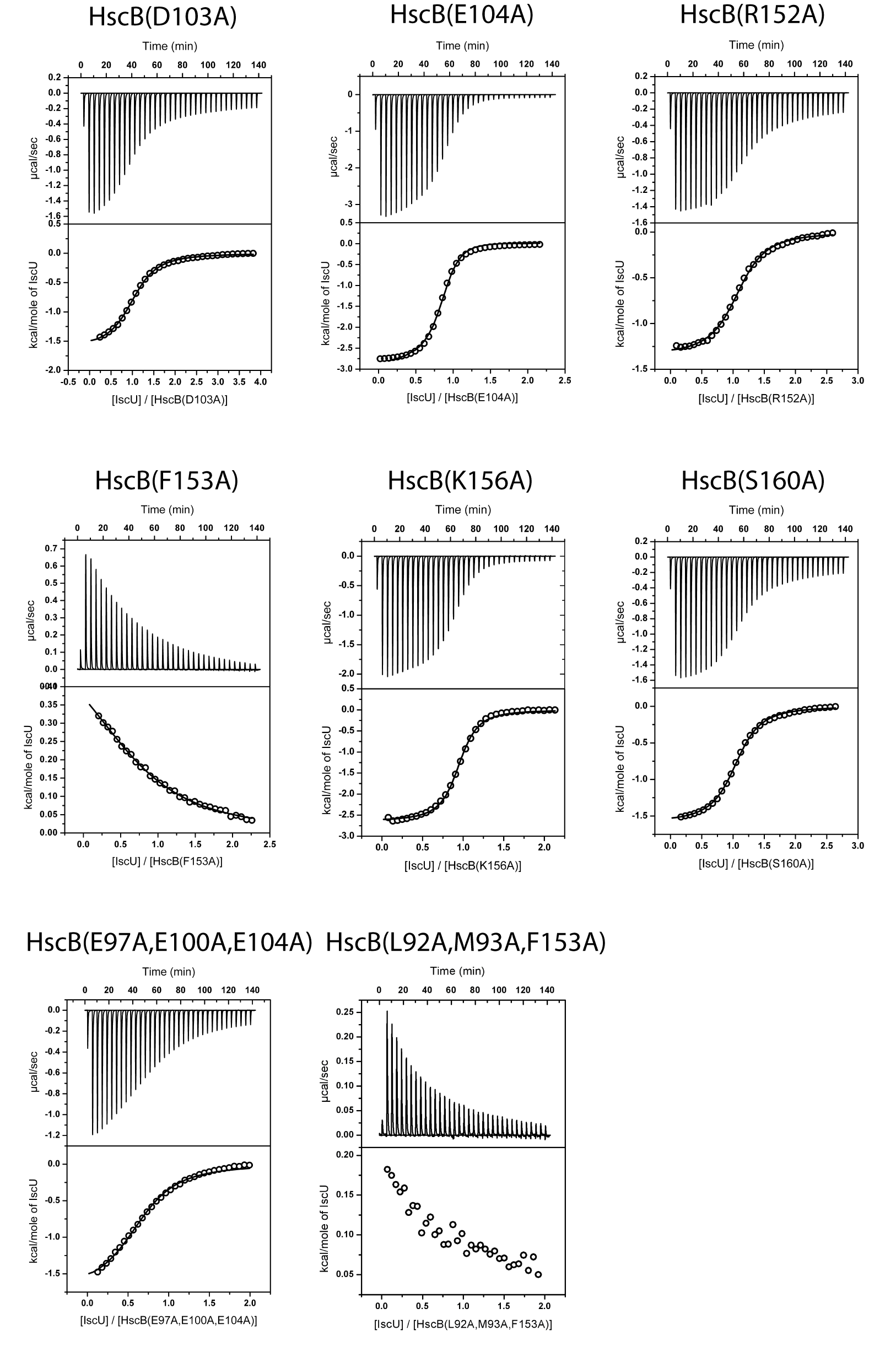
**
