# Supplementary material for: Three hydrophobic amino acids in Escherichia coli HscB make the greatest contribution to the stability of the HscB-IscU complex
Source: BMC Biochem. 2011 Jan 26;12:3. doi: 10.1186/1471-2091-12-3 (PMC3040723; doi:10.1186/1471-2091-12-3)

**Figure S3 −** **^15^N-HSQC spectrum of wild-type HscB, HscB(D103A), HscB(E100A), and HscB(L96A)**

Samples contained 0.2 mM [*U*-^15^N]-HscB in 20 mM Tris·HCl pH 7.5, 10 mM DTT, 0.1 mM DSS, and 7 % D_2_O. Each spectrum was recorded at 40°C on a Bruker DMX-750 Avance spectrometer equipped with a *z*-axis gradient cryoprobe. Peaks that are folded into the spectrum are colored in magenta.


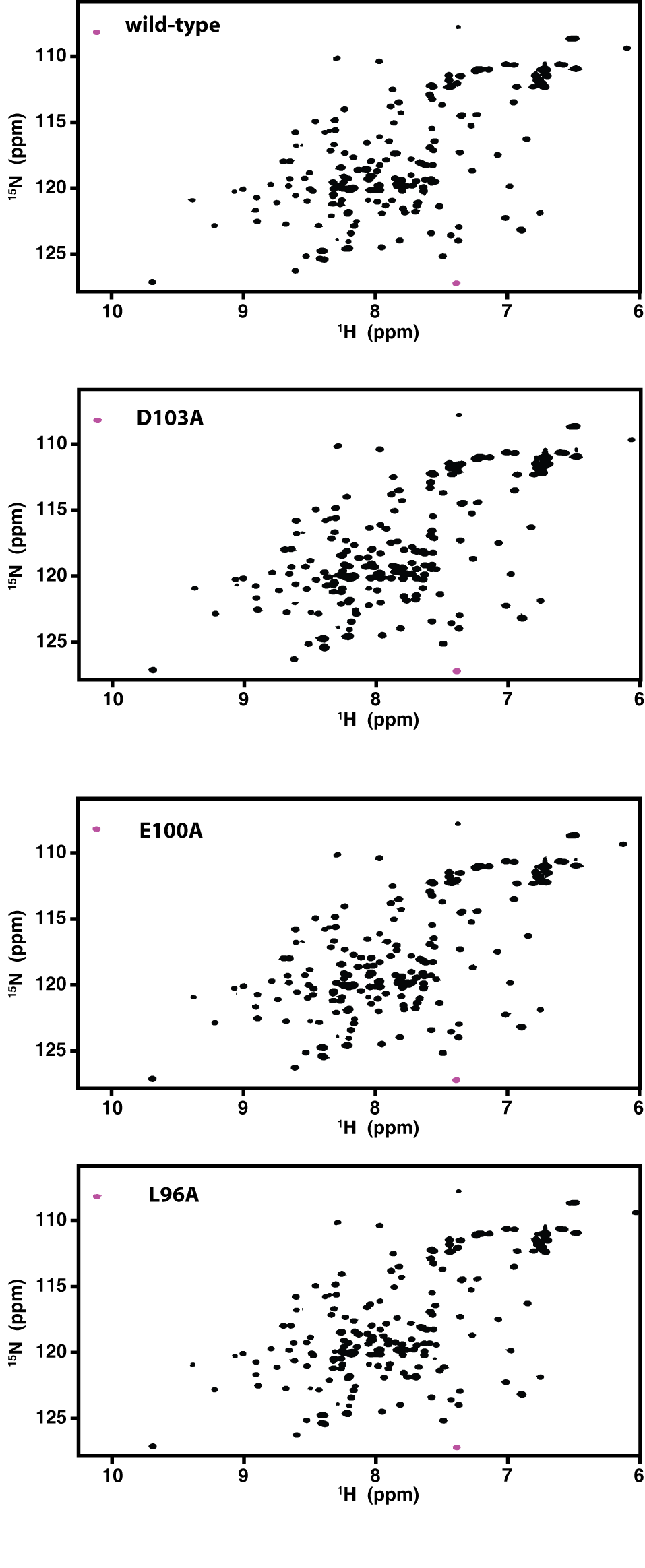

Supplement: Additional File 5 — 15N-HSQC spectra of wild-type HscB, HscB(D103A), HscB(E100A), and HscB(L96A) Samples contained 0.2 mM [U-15N]-HscB in 20 mM Tris-HCl pH 7.5, 10 mM DTT, 0.1 mM DSS, and 7 % D2O. Each spectrum was recorded at 40 °C on a Bruker DMX-750 Avance spectrometer equipped with a z-axis gradient cryoprobe. Peaks that are folded into the spectrum are colored in magenta. [file 1471-2091-12-3-S5.DOC]
