# Supplementary material for: Three hydrophobic amino acids in Escherichia coli HscB make the greatest contribution to the stability of the HscB-IscU complex
Source: BMC Biochem. 2011 Jan 26;12:3. doi: 10.1186/1471-2091-12-3 (PMC3040723; doi:10.1186/1471-2091-12-3)
Supplement: Additional File 6 — Chemical shift assignments for free and IscU-bound wild-type HscB 1H (δH) and 15N (δN) chemical shifts (in ppm) of assigned peaks in the 15N-HSQC spectrum of unbound HscB, and HscB in the presence of a six-fold molar excess of IscU ("IscU-bound HscB"). [file 1471-2091-12-3-S6.DOC]

**Table S3 – Chemical shift assignments for free and (apo-IscU)-bound wild-type HscB**

**^1^H (*δ*_H_) and ^15^N (*δ*_N_) chemical shifts (in ppm) of assigned peaks in the ^15^N-HSQC spectrum of unbound HscB, and HscB in the presence of a six-fold molar excess of apo-IscU (“IscU-bound HscB”).**

|  | unbound HscB | | IscU-bound HscB | |
| --- | --- | --- | --- | --- |
| Residue number | *δ*_H_ | *δ*_N_ | *δ*_H_ | *δ*_N_ |
| 3 | 8.604 | 116.771 | 8.592 | 116.763 |
| 4 | 8.384 | 115.773 | 8.370 | 115.758 |
| 5 | 9.070 | 120.268 | 9.067 | 120.267 |
| 6 | 8.205 | 124.044 | 8.200 | 124.034 |
| 7 | 7.230 | 114.410 | 7.232 | 114.417 |
| 8 | 8.289 | 110.136 | 8.287 | 110.118 |
| 9 | 8.322 | 121.183 | 8.329 | 121.194 |
| 11 | 8.219 | 124.592 | 8.224 | 124.633 |
| 13 | 9.386 | 120.920 | 9.459 | 120.957 |
| 14 | 8.698 | 117.980 | 8.733 | 117.934 |
| 15 | 7.578 | 123.413 | 7.581 | 123.283 |
| 18 | 8.395 | 124.749 | 8.391 | 124.731 |
| 19 | 7.817 | 123.957 | 7.813 | 123.924 |
| 21 | 7.885 | 113.801 | 7.884 | 113.803 |
| 22 | 7.698 | 121.772 | 7.689 | 121.757 |
| 24 | 8.659 | 119.826 | 8.658 | 119.833 |
| 26 | 7.692 | 119.455 | overlapped | |
| 27 | 8.265 | 121.218 | 8.258 | 121.162 |
| 28 | 8.340 | 117.146 | 8.336 | 117.142 |
| 29 | 7.362 | 117.299 | 7.361 | 117.291 |
| 32 | 7.015 | 122.256 | 7.012 | 122.230 |
| 34 | 10.332 | 121.376 | 10.324 | 121.387 |
| 35 | 8.132 | 118.620 | 8.131 | 118.626 |
| 36 | 7.588 | 116.896 | 7.587 | 116.903 |
| 37 | 7.372 | 123.970 | 7.372 | 123.956 |
| 39 | 7.971 | 110.385 | 7.971 | 110.384 |
| 40 | 8.607 | 115.780 | 8.602 | 115.783 |
| 43 | 7.801 | 119.391 | 7.796 | 119.370 |
| 45 | 8.317 | 120.038 | line broadened | |
| 46 | 7.790 | 121.540 | 7.786 | 121.530 |
| 47 | 7.515 | 121.362 | 7.515 | 121.351 |
| 48 | 7.795 | 120.200 | 7.794 | 120.177 |
| 49 | 7.966 | 120.143 | 7.969 | 120.162 |
| 50 | 8.317 | 120.486 | 8.316 | 120.484 |
| 51 | 8.307 | 114.843 | 8.299 | 114.827 |
| 52 | 7.953 | 124.477 | 7.954 | 124.466 |
| 53 | 8.033 | 117.953 | 8.028 | 117.933 |
| 54 | 8.255 | 120.918 | 8.245 | 120.945 |
| 55 | 7.967 | 118.257 | 7.963 | 118.246 |
| 56 | 8.039 | 120.150 | 8.033 | 120.177 |
| 57 | 8.412 | 124.752 | 8.411 | 124.722 |
| 58 | 8.204 | 119.262 | 8.194 | 119.260 |
| 59 | 7.861 | 115.048 | 7.852 | 115.065 |
| 60 | 7.495 | 113.685 | 7.503 | 113.727 |
| 61 | 7.806 | 114.286 | 7.808 | 114.299 |
| 62 | 7.969 | 116.109 | 7.950 | 116.091 |
| 69 | 8.315 | 116.673 | 8.328 | 116.612 |
| 70 | 8.909 | 121.669 | 8.947 | 121.905 |
| 71 | 8.613 | 122.065 | line broadened | |
| 72 | 8.232 | 117.327 | 8.226 | 117.250 |
| 74 | 7.367 | 122.943 | 7.239 | 122.581 |
| 75 | 7.274 | 115.266 | 7.361 | 115.497 |
| 76 | 7.387 | 127.201 | 7.330 | 126.818 |
| 77 | 7.636 | 119.929 | 7.563 | 119.573 |
| 78 | 8.431 | 122.835 | line broadened | |
| 79 | 8.389 | 125.422 | line broadened | |
| 81 | 7.590 | 112.918 | line broadened | |
| 82 | 8.143 | 122.514 | line broadened | |
| 86 | 8.161 | 122.866 | line broadened | |
| 90 | 8.203 | 124.553 | 8.276 | 123.982 |
| 91 | 7.815 | 119.583 | line broadened | |
| 92 | 7.839 | 119.696 | line broadened | |
| 94 | 7.685 | 121.301 | line broadened | |
| 95 | 7.896 | 116.440 | line broadened | |
| 96 | 7.572 | 119.578 | line broadened | |
| 100 | 8.322 | 119.547 | line broadened | |
| 101 | 7.922 | 121.307 | 7.973 | 122.227 |
| 102 | 7.832 | 119.853 | line broadened | |
| 103 | 7.987 | 118.711 | line broadened | |
| 104 | 7.875 | 120.941 | line broadened | |
| 105 | 8.334 | 122.097 | 8.386 | 122.117 |
| 106 | 8.497 | 120.055 | 8.519 | 120.211 |
| 107 | 7.919 | 117.856 | 8.010 | 118.168 |
| 108 | 7.853 | 119.239 | 7.814 | 119.036 |
| 109 | 8.066 | 116.548 | 8.048 | 116.905 |
| 110 | 6.853 | 116.278 | 6.895 | 116.180 |
| 111 | 8.607 | 126.251 | 8.569 | 126.112 |
| 112 | 8.186 | 123.430 | 8.188 | 123.511 |
| 115 | 8.240 | 119.543 | 8.221 | 119.656 |
| 116 | 7.570 | 113.257 | 7.518 | 113.189 |
| 117 | 8.526 | 125.153 | 8.531 | 125.222 |
| 118 | 8.793 | 119.712 | 8.863 | 119.707 |
| 120 | 7.985 | 121.037 | 7.931 | 120.927 |
| 121 | 8.475 | 120.223 | 8.413 | 120.317 |
| 122 | 8.518 | 120.991 | 8.603 | 121.093 |
| 123 | 7.719 | 119.795 | overlapped | |
| 124 | 8.247 | 120.015 | 8.243 | 119.988 |
| 125 | 8.747 | 121.093 | 8.746 | 120.894 |
| 126 | 8.900 | 120.715 | overlapped | |
| 127 | 8.304 | 115.603 | 8.268 | 115.893 |
| 128 | 7.776 | 121.857 | line broadened | |
| 129 | 9.220 | 122.840 | 9.237 | 122.215 |
| 130 | 7.559 | 117.129 | line broadened | |
| 131 | 6.982 | 119.850 | 6.940 | 120.020 |
| 133 | 7.730 | 117.789 | 7.795 | 117.900 |
| 134 | 7.267 | 118.682 | 7.315 | 118.733 |
| 136 | 8.500 | 118.832 | 8.455 | 118.995 |
| 137 | 8.677 | 122.724 | 8.748 | 122.777 |
| 138 | 7.573 | 115.469 | 7.575 | 115.566 |
| 140 | 7.871 | 112.504 | 7.813 | 112.039 |
| 141 | 6.752 | 121.874 | 6.768 | 121.904 |
| 142 | 8.456 | 114.935 | 8.497 | 115.079 |
| 143 | 7.430 | 123.572 | 7.454 | 123.811 |
| 144 | 7.729 | 118.996 | 7.751 | 118.944 |
| 145 | 8.240 | 119.109 | 8.264 | 118.963 |
| 146 | 7.075 | 117.486 | 7.082 | 117.230 |
| 147 | 8.166 | 117.665 | 8.123 | 117.280 |
| 148 | 8.897 | 122.525 | 8.835 | 122.732 |
| 149 | 7.765 | 119.822 | line broadened | |
| 150 | 7.547 | 119.504 | line broadened | |
| 151 | 8.605 | 120.563 | 8.700 | 121.364 |
| 152 | 8.649 | 119.290 | line broadened | |
| 153 | 7.579 | 119.297 | line broadened | |
| 154 | 8.282 | 119.813 | line broadened | |
| 155 | 9.004 | 120.077 | overlapped | |
| 156 | 7.625 | 119.337 | line broadened | |
| 157 | 7.951 | 121.908 | line broadened | |
| 158 | 8.537 | 119.253 | 8.514 | 119.116 |
| 159 | 8.236 | 114.020 | 8.330 | 114.086 |
| 161 | 8.420 | 125.343 | 8.383 | 125.217 |
| 162 | 8.648 | 117.961 | 8.592 | 117.872 |
| 164 | 7.677 | 121.086 | 7.661 | 121.137 |
| 165 | 8.005 | 120.131 | 7.994 | 120.213 |
| 166 | 7.551 | 116.437 | 7.525 | 116.239 |
| 167 | 7.566 | 118.255 | 7.561 | 118.210 |
| 169 | 7.616 | 118.284 | 7.614 | 118.192 |
| 170 | 7.635 | 120.221 | 7.631 | 120.161 |
| 171 | 7.490 | 125.164 | 7.496 | 125.171 |
