# Supplementary material for: Three hydrophobic amino acids in Escherichia coli HscB make the greatest contribution to the stability of the HscB-IscU complex
Source: BMC Biochem. 2011 Jan 26;12:3. doi: 10.1186/1471-2091-12-3 (PMC3040723; doi:10.1186/1471-2091-12-3)
Supplement: Additional File 7 — Chemical shift assignments for free and IscU-bound HscB(D103A) 1H (δH) and 15N (δN) chemical shifts (in ppm) of assigned peaks in the 15N-HSQC spectrum of unbound HscB(D103A), and HscB(D103A) in the presence of a six-fold molar excess of IscU ["IscU-bound HscB(D103A)"]. [file 1471-2091-12-3-S7.DOC]

**Table S4 – Chemical shift assignments for free and (apo-IscU)-bound HscB(D103A)**

**^1^H (*δ*_H_) and ^15^N (*δ*_N_) chemical shifts (in ppm) of assigned peaks in the ^15^N-HSQC spectrum of unbound HscB(D103A), and HscB(D103A) in the presence of a six-fold molar excess of apo-IscU [“IscU-bound HscB(D103A)”].**

|  | unbound HscB(D103A) | | IscU-bound HscB(D103A) | |
| --- | --- | --- | --- | --- |
| Residue number | *δ*_H_ | *δ*_N_ | *δ*_H_ | *δ*_N_ |
| 3 | 8.603 | 116.766 | 8.593 | 116.771 |
| 4 | 8.384 | 115.776 | 8.372 | 115.797 |
| 5 | 9.069 | 120.256 | 9.066 | 120.247 |
| 6 | 8.203 | 124.040 | 8.194 | 124.043 |
| 7 | 7.228 | 114.402 | 7.222 | 114.375 |
| 8 | 8.287 | 110.125 | 8.284 | 110.113 |
| 9 | 8.322 | 121.176 | 8.322 | 121.173 |
| 13 | 9.375 | 120.915 | 9.454 | 120.947 |
| 14 | 8.694 | 117.986 | 8.728 | 117.950 |
| 15 | 7.577 | 123.423 | 7.579 | 123.316 |
| 18 | 8.396 | 124.758 | overlapped | |
| 19 | 7.816 | 123.953 | 7.810 | 123.922 |
| 20 | 8.062 | 118.569 | 8.061 | 118.584 |
| 21 | 7.885 | 113.798 | 7.883 | 113.815 |
| 22 | 7.699 | 121.771 | 7.693 | 121.753 |
| 24 | 8.657 | 119.825 | 8.654 | 119.824 |
| 25 | 7.840 | 117.378 | 7.837 | 117.371 |
| 26 | 7.691 | 119.454 | 7.686 | 119.446 |
| 27 | 8.263 | 121.209 | 8.257 | 121.170 |
| 28 | 8.339 | 117.145 | 8.333 | 117.149 |
| 29 | 7.361 | 117.299 | 7.360 | 117.295 |
| 32 | 7.015 | 122.251 | 7.016 | 122.207 |
| 34 | 10.326 | 121.370 | 10.305 | 121.360 |
| 35 | 8.131 | 118.622 | 8.127 | 118.627 |
| 36 | 7.588 | 116.898 | 7.590 | 116.918 |
| 37 | 7.373 | 123.966 | 7.374 | 123.956 |
| 39 | 7.972 | 110.383 | 7.970 | 110.376 |
| 40 | 8.607 | 115.778 | 8.600 | 115.786 |
| 43 | 7.800 | 119.386 | 7.793 | 119.363 |
| 46 | 7.789 | 121.536 | 7.785 | 121.526 |
| 47 | 7.515 | 121.364 | 7.517 | 121.350 |
| 48 | 7.796 | 120.193 | 7.793 | 120.171 |
| 50 | 8.317 | 120.489 | 8.314 | 120.483 |
| 51 | 8.306 | 114.846 | 8.298 | 114.837 |
| 52 | 7.954 | 124.476 | 7.954 | 124.451 |
| 53 | 8.031 | 117.946 | 8.024 | 117.913 |
| 54 | 8.254 | 120.919 | 8.242 | 120.908 |
| 55 | 7.967 | 118.256 | 7.962 | 118.250 |
| 56 | 8.041 | 120.142 | 8.034 | 120.133 |
| 57 | 8.411 | 124.754 | overlapped | |
| 58 | 8.204 | 119.263 | 8.193 | 119.259 |
| 59 | 7.861 | 115.044 | 7.855 | 115.059 |
| 60 | 7.494 | 113.685 | 7.499 | 113.712 |
| 61 | 7.805 | 114.284 | 7.811 | 114.308 |
| 62 | 7.968 | 116.105 | 7.948 | 116.100 |
| 69 | 8.314 | 116.666 | 8.326 | 116.588 |
| 70 | 8.907 | 121.660 | 8.944 | 121.883 |
| 71 | 8.619 | 122.066 | line broadened | |
| 72 | 8.230 | 117.303 | 8.226 | 117.281 |
| 74 | 7.367 | 122.951 | 7.239 | 122.556 |
| 75 | 7.275 | 115.256 | 7.363 | 115.452 |
| 76 | 7.388 | 127.198 | 7.335 | 126.825 |
| 77 | 7.632 | 119.872 | 7.559 | 119.580 |
| 78 | 8.436 | 122.835 | line broadened | |
| 81 | 7.587 | 112.901 | line broadened | |
| 82 | 8.155 | 122.570 | line broadened | |
| 86 | 8.150 | 122.844 | line broadened | |
| 94 | 7.696 | 121.453 | line broadened | |
| 95 | 7.924 | 116.397 | line broadened | |
| 96 | 7.579 | 119.458 | line broadened | |
| 105 | 8.267 | 122.120 | overlapped | |
| 108 | 7.841 | 119.313 | overlapped | |
| 109 | 8.051 | 116.338 | 8.049 | 116.561 |
| 110 | 6.826 | 116.295 | 6.835 | 116.181 |
| 111 | 8.622 | 126.303 | 8.575 | 126.184 |
| 112 | 8.187 | 123.444 | 8.183 | 123.442 |
| 115 | 8.252 | 119.607 | 8.235 | 119.621 |
| 116 | 7.592 | 113.294 | 7.511 | 113.166 |
| 117 | 8.513 | 125.137 | 8.502 | 125.116 |
| 118 | 8.787 | 119.732 | 8.858 | 119.674 |
| 120 | 7.991 | 121.076 | 7.911 | 120.824 |
| 121 | 8.456 | 120.281 | 8.400 | 120.251 |
| 122 | 8.526 | 120.964 | 8.605 | 121.132 |
| 123 | 7.732 | 119.812 | overlapped | |
| 124 | 8.247 | 120.006 | 8.240 | 120.004 |
| 125 | 8.737 | 121.078 | 8.750 | 120.915 |
| 126 | 8.912 | 120.742 | overlapped | |
| 127 | 8.307 | 115.598 | 8.258 | 115.836 |
| 128 | 7.767 | 121.857 | 7.716 | 121.430 |
| 129 | 9.219 | 122.837 | 9.232 | 122.217 |
| 130 | 7.560 | 117.125 | line broadened | |
| 131 | 6.979 | 119.846 | 6.934 | 119.981 |
| 132 | 8.034 | 119.042 | overlapped | |
| 133 | 7.729 | 117.790 | 7.785 | 117.877 |
| 134 | 7.266 | 118.680 | 7.308 | 118.715 |
| 136 | 8.499 | 118.833 | 8.451 | 118.961 |
| 137 | 8.677 | 122.727 | 8.737 | 122.758 |
| 138 | 7.571 | 115.457 | 7.574 | 115.558 |
| 140 | 7.869 | 112.493 | 7.812 | 112.066 |
| 141 | 6.753 | 121.869 | 6.769 | 121.930 |
| 142 | 8.457 | 114.954 | 8.495 | 115.098 |
| 143 | 7.430 | 123.562 | 7.456 | 123.799 |
| 144 | 7.729 | 118.991 | 7.753 | 118.977 |
| 145 | 8.240 | 119.112 | 8.260 | 119.002 |
| 146 | 7.072 | 117.488 | 7.077 | 117.238 |
| 147 | 8.166 | 117.655 | 8.134 | 117.297 |
| 148 | 8.898 | 122.537 | 8.847 | 122.754 |
| 149 | 7.769 | 119.821 | overlapped | |
| 150 | 7.546 | 119.474 | line broadened | |
| 151 | 8.613 | 120.609 | 8.723 | 121.345 |
| 152 | 8.640 | 119.304 | line broadened | |
| 153 | 7.576 | 119.142 | line broadened | |
| 155 | 9.006 | 120.171 | overlapped | |
| 156 | 7.631 | 119.178 | 7.592 | 118.161 |
| 157 | 7.965 | 122.011 | line broadened | |
| 158 | 8.535 | 119.288 | 8.508 | 119.106 |
| 159 | 8.222 | 113.980 | 8.360 | 114.089 |
| 162 | 8.643 | 117.948 | 8.596 | 117.903 |
| 164 | 7.646 | 120.904 | 7.637 | 120.968 |
| 166 | 7.577 | 116.548 | 7.552 | 116.370 |
| 167 | 7.570 | 118.228 | 7.562 | 118.199 |
| 170 | 7.644 | 120.251 | 7.635 | 120.182 |
| 171 | 7.492 | 125.132 | 7.499 | 125.136 |
