# Supplementary material for: Three hydrophobic amino acids in Escherichia coli HscB make the greatest contribution to the stability of the HscB-IscU complex
Source: BMC Biochem. 2011 Jan 26;12:3. doi: 10.1186/1471-2091-12-3 (PMC3040723; doi:10.1186/1471-2091-12-3)
Supplement: Additional File 8 — Chemical shift assignments for free and IscU-bound HscB(E100A) 1H (δH) and 15N (δN) chemical shifts (in ppm) of assigned peaks in the 15N-HSQC spectrum of unbound HscB(E100A), and HscB(E100A) in the presence of a six-fold molar excess of IscU ["IscU-bound HscB(E100A)"]. [file 1471-2091-12-3-S8.DOC]

**Table S5 – Chemical shift assignments for free and (apo-IscU)-bound HscB(E100A)**

**^1^H (*δ*_H_) and ^15^N (*δ*_N_) chemical shifts (in ppm) of assigned peaks in the ^15^N-HSQC spectrum of unbound HscB(E100A), and HscB(E100A) in the presence of a six-fold molar excess of apo-IscU [“IscU-bound HscB(E100A)”].**

|  | unbound HscB(E100A) | | IscU-bound HscB(E100A) | |
| --- | --- | --- | --- | --- |
| Residue number | *δ*_H_ | *δ*_N_ | *δ*_H_ | *δ*_N_ |
| 3 | 8.602 | 116.769 | 8.594 | 116.771 |
| 4 | 8.384 | 115.782 | 8.375 | 115.781 |
| 5 | 9.070 | 120.263 | 9.070 | 120.278 |
| 6 | 8.201 | 124.040 | 8.198 | 124.050 |
| 7 | 7.225 | 114.393 | 7.223 | 114.380 |
| 8 | 8.287 | 110.129 | 8.286 | 110.107 |
| 9 | 8.320 | 121.172 | 8.324 | 121.177 |
| 11 | 8.216 | 124.590 | 8.221 | 124.601 |
| 13 | 9.378 | 120.912 | 9.436 | 120.960 |
| 14 | 8.693 | 117.984 | 8.721 | 117.952 |
| 15 | 7.576 | 123.418 | 7.580 | 123.335 |
| 18 | 8.391 | 124.761 | overlapped | |
| 19 | 7.815 | 123.954 | 7.812 | 123.936 |
| 20 | 8.061 | 118.567 | 8.061 | 118.596 |
| 21 | 7.884 | 113.798 | 7.883 | 113.817 |
| 22 | 7.697 | 121.773 | 7.694 | 121.763 |
| 23 | 7.969 | 119.730 | overlapped | |
| 24 | 8.656 | 119.825 | 8.656 | 119.828 |
| 25 | 7.838 | 117.371 | 7.839 | 117.380 |
| 26 | 7.690 | 119.452 | overlapped | |
| 27 | 8.262 | 121.209 | 8.259 | 121.185 |
| 28 | 8.338 | 117.144 | 8.336 | 117.145 |
| 29 | 7.360 | 117.297 | 7.360 | 117.293 |
| 32 | 7.015 | 122.250 | 7.015 | 122.227 |
| 34 | 10.323 | 121.374 | 10.317 | 121.359 |
| 35 | 8.130 | 118.620 | 8.128 | 118.627 |
| 36 | 7.588 | 116.897 | 7.589 | 116.910 |
| 37 | 7.372 | 123.966 | 7.374 | 123.961 |
| 39 | 7.970 | 110.388 | 7.971 | 110.379 |
| 40 | 8.606 | 115.780 | 8.602 | 115.785 |
| 43 | 7.799 | 119.383 | 7.795 | 119.356 |
| 46 | 7.788 | 121.536 | 7.787 | 121.531 |
| 47 | 7.515 | 121.361 | 7.517 | 121.354 |
| 48 | 7.795 | 120.195 | 7.795 | 120.170 |
| 49 | 7.965 | 120.136 | 7.969 | 120.140 |
| 50 | 8.316 | 120.490 | 8.316 | 120.489 |
| 51 | 8.305 | 114.843 | 8.302 | 114.841 |
| 52 | 7.952 | 124.474 | 7.955 | 124.452 |
| 53 | 8.029 | 117.945 | 8.027 | 117.939 |
| 54 | 8.252 | 120.923 | 8.246 | 120.907 |
| 55 | 7.966 | 118.254 | 7.963 | 118.239 |
| 56 | 8.039 | 120.140 | 8.038 | 120.154 |
| 57 | 8.410 | 124.750 | overlapped | |
| 58 | 8.203 | 119.264 | 8.194 | 119.259 |
| 59 | 7.861 | 115.045 | 7.857 | 115.061 |
| 60 | 7.493 | 113.682 | 7.500 | 113.703 |
| 61 | 7.804 | 114.283 | 7.805 | 114.292 |
| 62 | 7.968 | 116.111 | 7.955 | 116.106 |
| 69 | 8.314 | 116.667 | 8.325 | 116.622 |
| 70 | 8.907 | 121.671 | 8.936 | 121.844 |
| 71 | 8.612 | 122.063 | 8.616 | 122.271 |
| 72 | 8.231 | 117.316 | 8.233 | 117.288 |
| 74 | 7.367 | 122.948 | 7.276 | 122.683 |
| 75 | 7.272 | 115.243 | 7.334 | 115.422 |
| 76 | 7.387 | 127.208 | 7.346 | 126.925 |
| 77 | 7.634 | 119.899 | 7.583 | 119.669 |
| 78 | 8.426 | 122.821 | line broadened | |
| 79 | 8.397 | 125.420 | line broadened | |
| 81 | 7.588 | 112.927 | line broadened | |
| 82 | 8.159 | 122.572 | line broadened | |
| 86 | 8.163 | 122.880 | line broadened | |
| 94 | 7.683 | 121.344 | line broadened | |
| 95 | 7.910 | 116.705 | line broadened | |
| 103 | 8.016 | 118.538 | line broadened | |
| 105 | 8.296 | 122.099 | 8.365 | 122.132 |
| 106 | 8.505 | 120.008 | 8.500 | 120.108 |
| 107 | 7.884 | 117.813 | 7.943 | 117.990 |
| 108 | 7.848 | 119.246 | 7.822 | 119.073 |
| 109 | 8.060 | 116.522 | 8.051 | 116.808 |
| 110 | 6.843 | 116.282 | 6.879 | 116.216 |
| 111 | 8.611 | 126.268 | 8.577 | 126.156 |
| 112 | 8.185 | 123.420 | 8.190 | 123.471 |
| 115 | 8.238 | 119.526 | 8.224 | 119.599 |
| 116 | 7.569 | 113.238 | 7.528 | 113.175 |
| 117 | 8.528 | 125.128 | 8.526 | 125.148 |
| 118 | 8.787 | 119.717 | 8.839 | 119.710 |
| 120 | 7.982 | 121.038 | 7.946 | 120.901 |
| 121 | 8.469 | 120.273 | 8.429 | 120.294 |
| 122 | 8.513 | 120.985 | 8.580 | 121.101 |
| 123 | 7.719 | 119.791 | overlapped | |
| 124 | 8.245 | 120.011 | 8.244 | 119.989 |
| 125 | 8.740 | 121.092 | 8.743 | 121.005 |
| 126 | 8.895 | 120.726 | 9.005 | 120.644 |
| 127 | 8.301 | 115.600 | 8.283 | 115.806 |
| 128 | 7.781 | 121.858 | line broadened | |
| 129 | 9.217 | 122.855 | 9.232 | 122.387 |
| 130 | 7.552 | 117.130 | line broadened | |
| 131 | 6.979 | 119.838 | 6.949 | 119.963 |
| 133 | 7.728 | 117.794 | 7.772 | 117.862 |
| 134 | 7.263 | 118.678 | 7.295 | 118.706 |
| 136 | 8.499 | 118.835 | 8.471 | 118.967 |
| 137 | 8.675 | 122.727 | 8.722 | 122.757 |
| 138 | 7.570 | 115.460 | 7.575 | 115.534 |
| 140 | 7.869 | 112.501 | 7.830 | 112.184 |
| 141 | 6.750 | 121.872 | 6.763 | 121.911 |
| 142 | 8.454 | 114.952 | 8.487 | 115.058 |
| 143 | 7.428 | 123.542 | 7.448 | 123.744 |
| 144 | 7.728 | 119.011 | 7.746 | 118.960 |
| 145 | 8.239 | 119.110 | 8.255 | 119.010 |
| 146 | 7.076 | 117.488 | 7.082 | 117.284 |
| 147 | 8.166 | 117.701 | 8.141 | 117.383 |
| 148 | 8.893 | 122.540 | 8.853 | 122.665 |
| 149 | 7.759 | 119.825 | overlapped | |
| 150 | 7.539 | 119.567 | line broadened | |
| 151 | 8.596 | 120.546 | 8.671 | 121.059 |
| 152 | 8.665 | 119.297 | line broadened | |
| 153 | 7.580 | 119.278 | line broadened | |
| 154 | 8.294 | 119.824 | line broadened | |
| 155 | 9.000 | 120.087 | 9.083 | 120.548 |
| 156 | 7.627 | 119.322 | 7.581 | 118.482 |
| 157 | 7.975 | 122.000 | line broadened | |
| 158 | 8.535 | 119.263 | 8.501 | 119.129 |
| 159 | 8.234 | 114.036 | 8.331 | 114.103 |
| 161 | 8.403 | 125.378 | overlapped | |
| 162 | 8.653 | 117.981 | 8.611 | 117.929 |
| 164 | 7.664 | 121.010 | 7.660 | 121.086 |
| 165 | 7.995 | 120.120 | 7.989 | 120.185 |
| 166 | 7.558 | 116.458 | 7.535 | 116.296 |
| 167 | 7.565 | 118.253 | 7.559 | 118.211 |
| 169 | 7.619 | 118.293 | 7.616 | 118.205 |
| 170 | 7.635 | 120.226 | 7.632 | 120.170 |
| 171 | 7.490 | 125.154 | 7.497 | 125.153 |
