# Supplementary material for: Three hydrophobic amino acids in Escherichia coli HscB make the greatest contribution to the stability of the HscB-IscU complex
Source: BMC Biochem. 2011 Jan 26;12:3. doi: 10.1186/1471-2091-12-3 (PMC3040723; doi:10.1186/1471-2091-12-3)
Supplement: Additional File 9 — Chemical shift assignments for free and IscU-bound HscB(L96A) 1H (δH) and 15N (δN) chemical shifts (in ppm) of assigned peaks in the 15N-HSQC spectrum of unbound HscB(L96A), and HscB(L96A) in the presence of a six-fold molar excess of IscU ["IscU-bound HscB(L96A)"]. [file 1471-2091-12-3-S9.DOC]

**Table S6 – Chemical shift assignments for free and (apo-IscU)-bound HscB(L96A)**

**^1^H (*δ*_H_) and ^15^N (*δ*_N_) chemical shifts (in ppm) of assigned peaks in the ^15^N-HSQC spectrum of unbound HscB(L96A), and HscB(L96A) in the presence of a six-fold molar excess of apo-IscU [“IscU-bound HscB(L96A)”].**

|  | unbound HscB(L96A) | | IscU-bound HscB(L96A) | |
| --- | --- | --- | --- | --- |
| Residue number | *δ*_H_ | *δ*_N_ | *δ*_H_ | *δ*_N_ |
| 3 | 8.603 | 116.770 | 8.596 | 116.768 |
| 4 | 8.384 | 115.776 | 8.377 | 115.772 |
| 5 | 9.069 | 120.265 | 9.055 | 120.269 |
| 6 | 8.203 | 124.041 | 8.198 | 124.043 |
| 7 | 7.226 | 114.402 | 7.224 | 114.381 |
| 8 | 8.288 | 110.128 | 8.285 | 110.114 |
| 9 | 8.322 | 121.180 | 8.320 | 121.171 |
| 11 | 8.218 | 124.611 | 8.219 | 124.594 |
| 13 | 9.389 | 120.917 | 9.414 | 120.934 |
| 14 | 8.699 | 117.979 | 8.707 | 117.958 |
| 15 | 7.577 | 123.402 | 7.577 | 123.375 |
| 18 | 8.396 | 124.758 | overlapped | |
| 19 | 7.816 | 123.956 | 7.811 | 123.929 |
| 21 | 7.884 | 113.800 | 7.882 | 113.812 |
| 22 | 7.697 | 121.832 | 7.695 | 121.771 |
| 24 | 8.657 | 119.824 | 8.655 | 119.824 |
| 25 | 7.838 | 117.372 | 7.838 | 117.376 |
| 26 | 7.690 | 119.454 | 7.688 | 119.451 |
| 27 | 8.263 | 121.214 | 8.259 | 121.181 |
| 28 | 8.339 | 117.143 | 8.335 | 117.143 |
| 29 | 7.360 | 117.294 | 7.360 | 117.292 |
| 32 | 7.014 | 122.248 | 7.013 | 122.229 |
| 34 | 10.326 | 121.377 | 10.317 | 121.341 |
| 35 | 8.131 | 118.622 | 8.128 | 118.618 |
| 36 | 7.588 | 116.897 | 7.588 | 116.906 |
| 37 | 7.372 | 123.964 | 7.372 | 123.956 |
| 39 | 7.971 | 110.384 | 7.969 | 110.377 |
| 40 | 8.606 | 115.779 | 8.602 | 115.778 |
| 43 | 7.798 | 119.381 | 7.795 | 119.368 |
| 46 | 7.788 | 121.538 | 7.786 | 121.534 |
| 47 | 7.515 | 121.360 | 7.516 | 121.353 |
| 48 | 7.795 | 120.194 | 7.794 | 120.168 |
| 49 | 7.965 | 120.140 | 7.965 | 120.134 |
| 50 | 8.317 | 120.487 | 8.315 | 120.480 |
| 51 | 8.307 | 114.841 | 8.301 | 114.840 |
| 52 | 7.953 | 124.473 | 7.954 | 124.457 |
| 53 | 8.030 | 117.947 | 8.026 | 117.923 |
| 54 | 8.253 | 120.916 | 8.244 | 120.914 |
| 55 | 7.966 | 118.255 | 7.964 | 118.259 |
| 56 | 8.039 | 120.148 | 8.036 | 120.145 |
| 57 | 8.411 | 124.752 | overlapped | |
| 58 | 8.203 | 119.259 | 8.197 | 119.259 |
| 59 | 7.860 | 115.044 | 7.857 | 115.053 |
| 60 | 7.494 | 113.687 | 7.496 | 113.696 |
| 61 | 7.806 | 114.287 | 7.800 | 114.279 |
| 62 | 7.968 | 116.106 | 7.960 | 116.128 |
| 69 | 8.316 | 116.677 | 8.316 | 116.637 |
| 70 | 8.908 | 121.664 | 8.920 | 121.746 |
| 71 | 8.614 | 122.072 | 8.611 | 122.162 |
| 72 | 8.231 | 117.328 | 8.228 | 117.321 |
| 74 | 7.363 | 122.927 | 7.321 | 122.812 |
| 75 | 7.276 | 115.258 | 7.302 | 115.334 |
| 76 | 7.386 | 127.192 | 7.367 | 127.065 |
| 77 | 7.631 | 119.912 | 7.608 | 119.836 |
| 78 | 8.430 | 122.835 | line broadened | |
| 79 | 8.387 | 125.431 | 8.337 | 125.198 |
| 80 | 8.197 | 121.783 | 8.191 | 121.302 |
| 81 | 7.582 | 112.893 | 7.518 | 112.278 |
| 82 | 8.155 | 122.551 | line broadened | |
| 86 | 8.168 | 122.868 | line broadened | |
| 90 | 8.218 | 124.611 | 8.238 | 124.303 |
| 101 | 7.905 | 121.210 | 7.909 | 121.379 |
| 104 | 7.895 | 120.874 | 7.867 | 120.861 |
| 105 | 8.353 | 122.097 | 8.377 | 122.094 |
| 106 | 8.486 | 120.070 | 8.484 | 120.149 |
| 107 | 7.921 | 117.817 | 7.958 | 117.917 |
| 108 | 7.853 | 119.215 | 7.841 | 119.146 |
| 109 | 8.068 | 116.567 | 8.062 | 116.702 |
| 110 | 6.850 | 116.275 | 6.863 | 116.234 |
| 111 | 8.601 | 126.248 | 8.592 | 126.187 |
| 112 | 8.186 | 123.415 | 8.185 | 123.465 |
| 114 | 8.210 | 121.919 | 8.204 | 121.840 |
| 115 | 8.240 | 119.540 | 8.217 | 119.552 |
| 116 | 7.563 | 113.253 | 7.540 | 113.211 |
| 117 | 8.525 | 125.143 | 8.525 | 125.147 |
| 118 | 8.795 | 119.709 | 8.818 | 119.689 |
| 120 | 7.982 | 121.012 | 7.961 | 120.941 |
| 121 | 8.474 | 120.217 | 8.457 | 120.230 |
| 122 | 8.521 | 121.010 | 8.548 | 121.057 |
| 123 | 7.711 | 119.774 | 7.689 | 119.650 |
| 125 | 8.749 | 121.113 | 8.753 | 121.060 |
| 126 | 8.907 | 120.718 | 8.957 | 120.665 |
| 127 | 8.299 | 115.623 | 8.285 | 115.718 |
| 128 | 7.759 | 121.860 | line broadened | |
| 129 | 9.223 | 122.811 | 9.228 | 122.583 |
| 130 | 7.570 | 117.139 | 7.631 | 117.226 |
| 131 | 6.976 | 119.851 | 6.965 | 119.913 |
| 133 | 7.734 | 117.796 | 7.756 | 117.842 |
| 134 | 7.270 | 118.689 | 7.290 | 118.723 |
| 136 | 8.495 | 118.842 | 8.479 | 118.901 |
| 137 | 8.681 | 122.723 | 8.703 | 122.729 |
| 138 | 7.573 | 115.469 | 7.574 | 115.504 |
| 140 | 7.869 | 112.495 | 7.850 | 112.367 |
| 141 | 6.752 | 121.863 | 6.756 | 121.876 |
| 142 | 8.456 | 114.945 | 8.469 | 114.983 |
| 143 | 7.431 | 123.583 | 7.436 | 123.669 |
| 144 | 7.729 | 118.993 | 7.733 | 118.975 |
| 145 | 8.240 | 119.086 | 8.243 | 119.042 |
| 146 | 7.072 | 117.483 | 7.072 | 117.384 |
| 147 | 8.167 | 117.609 | 8.156 | 117.473 |
| 148 | 8.892 | 122.518 | 8.868 | 122.593 |
| 149 | 7.768 | 119.797 | overlapped | |
| 150 | 7.541 | 119.421 | line broadened | |
| 151 | 8.616 | 120.624 | 8.655 | 120.937 |
| 152 | 8.623 | 119.261 | line broadened | |
| 153 | 7.591 | 119.278 | line broadened | |
| 155 | 9.007 | 120.075 | 9.043 | 120.270 |
| 156 | 7.633 | 119.304 | 7.603 | 118.935 |
| 157 | 7.937 | 121.902 | line broadened | |
| 158 | 8.524 | 119.236 | 8.511 | 119.164 |
| 159 | 8.259 | 114.033 | 8.298 | 114.075 |
| 161 | 8.409 | 125.356 | 8.405 | 125.314 |
| 162 | 8.642 | 117.968 | 8.617 | 117.946 |
| 163 | 8.057 | 119.332 | 8.081 | 119.336 |
| 164 | 7.675 | 121.083 | 7.674 | 121.124 |
| 165 | 8.002 | 120.128 | 8.006 | 120.169 |
| 166 | 7.547 | 116.417 | 7.531 | 116.329 |
| 167 | 7.563 | 118.249 | 7.561 | 118.240 |
| 169 | 7.614 | 118.273 | 7.612 | 118.230 |
| 170 | 7.632 | 120.214 | 7.630 | 120.171 |
| 171 | 7.489 | 125.165 | 7.493 | 125.148 |
