# Supplementary material for: Three hydrophobic amino acids in Escherichia coli HscB make the greatest contribution to the stability of the HscB-IscU complex
Source: BMC Biochem. 2011 Jan 26;12:3. doi: 10.1186/1471-2091-12-3 (PMC3040723; doi:10.1186/1471-2091-12-3)
Supplement: Additional File 10 — Combined chemical shift changes of free HscB(D103A), HscB(E100A), and HscB(L96A) relative to wild-type HscB Combined chemical shift changes are reported as the combination of changes in the proton (ΔδHAla) and nitrogen (ΔδNAla) dimensions according to ΔδHNAla = [(ΔδHAla)2 + ΔδNAla/6)2]1/2 [29]. [file 1471-2091-12-3-S10.DOC]

**Figure S3 – Combined chemical shift changes of free HscB(D103A), HscB(E100A), and HscB(L96A) relative to wild-type HscB**

Combined chemical shift changes are reported as the combination of changes in the proton (Δ*δ*_H_^Ala^) and nitrogen (Δ*δ*_N_^Ala^) dimensions according to Δ*δ*_HN_^Ala^ = [(Δ*δ*_H_^Ala^)^2^ + Δ*δ*_N_^Ala^/6)^2^]^1/2^ [29].

**
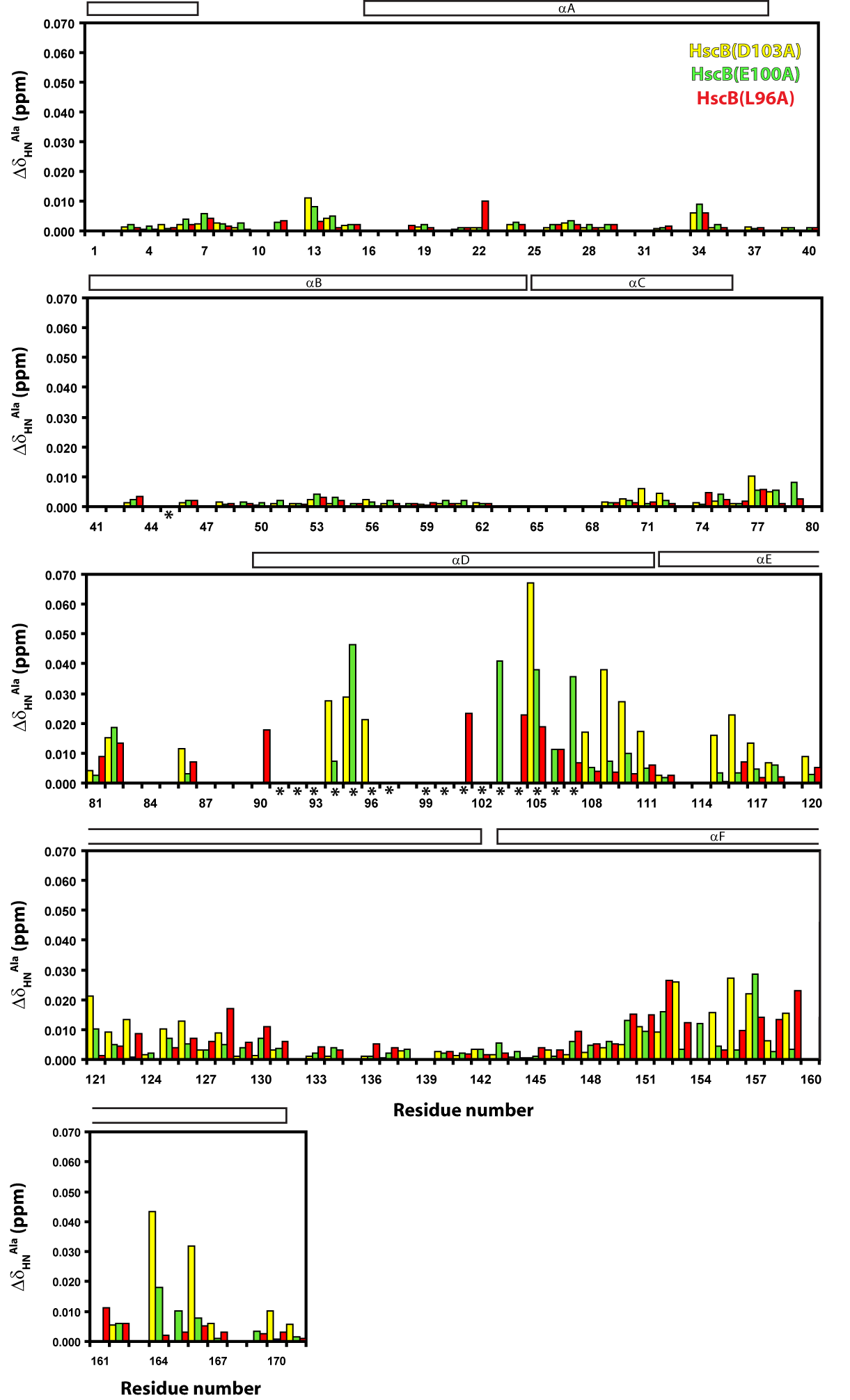
**
